# pH-Responsive N^C-Cyclometalated Iridium(III) Complexes: Synthesis, Photophysical Properties, Computational Results, and Bioimaging Application

**DOI:** 10.3390/molecules27010232

**Published:** 2021-12-30

**Authors:** Anastasia I. Solomatina, Daria O. Kozina, Vitaly V. Porsev, Sergey P. Tunik

**Affiliations:** Institute of Chemistry, St. Petersburg State University, Universitetskii Av., 26, 198504 St. Petersburg, Russia; kozina.d@yandex.ru

**Keywords:** orthometalated iridium(III) complexes, pH-dependent luminescence, phosphorescence lifetime imaging

## Abstract

Herein we report four [Ir(**N^C**)_2_(**L^L**)]^n+^, n = 0,1 complexes (**1**–**4**) containing cyclometallated **N^C** ligand (**N^CH** = 1-phenyl-2-(4-(pyridin-2-yl)phenyl)-1*H*-phenanthro[9,10-d]imidazole) and various bidentate **L^L** ligands (picolinic acid (**1**), 2,2′-bipyridine (**2**), [2,2′-bipyridine]-4,4′-dicarboxylic acid (**3**), and sodium 4,4′,4″,4‴-(1,2-phenylenebis(phosphanetriyl))tetrabenzenesulfonate (**4**). The **N^CH** ligand precursor and iridium complexes **1**–**4** were synthesized in good yield and characterized using chemical analysis, ESI mass spectrometry, and NMR spectroscopy. The solid-state structure of **2** was also determined by XRD analysis. The complexes display moderate to strong phosphorescence in the 550–670 nm range with the quantum yields up to 30% and lifetimes of the excited state up to 60 µs in deoxygenated solution. Emission properties of **1**–**4** and **N^CH** are strongly pH-dependent to give considerable variations in excitation and emission profiles accompanied by changes in emission efficiency and dynamics of the excited state. Density functional theory (DFT) and time-dependent density functional theory (TD DFT) calculations made it possible to assign the nature of emissive excited states in both deprotonated and protonated forms of these molecules. The complexes **3** and **4** internalize into living CHO-K1 cells, localize in cytoplasmic vesicles, primarily in lysosomes and acidified endosomes, and demonstrate relatively low toxicity, showing more than 80% cells viability up to the concentration of 10 µM after 24 h incubation. Phosphorescence lifetime imaging microscopy (PLIM) experiments in these cells display lifetime distribution, the conversion of which into pH values using calibration curves gives the magnitudes of this parameter compatible with the physiologically relevant interval of the cell compartments pH.

## 1. Introduction

Monitoring of pH in living cells is a rapidly growing field of optical sensor applications [1,2,3,4]. Acidity homeostasis is a crucial function of living organisms, the deviations of which from “normal” magnitudes is an indicator of pathology [5] or malignancy [6,7]. For example, it is well-known that the cancer cells’ pH increases with the simultaneous acidification of the extracellular environment compared to the healthy tissues [8,9,10]. Within the cell, pH also displays considerable inhomogeneity varying from the values of ca. 8.0 in mitochondria to 4.5 in lysosomes. The study of the physiological processes in these organelles requires real-time pH monitoring that, in turn, needs the sensors, which exhibit a pronounced analytical response to changes in acidity in the physiological range. In this respect, luminescent sensors are extremely convenient for these studies because of their high sensitivity and noninvasive sensing techniques.

Luminescent sensors may operate in two imaging modes: luminescent microscopy and fluorescence/phosphorescence lifetime imaging microscopy (FLIM/PLIM). The former usually measure emission intensity and/or wavelength that implies the presence of internal standard to quantify the sensory response. On the contrary, the use of sensors operating in FLIM/PLIM mode does not need an internal standard for the quantitative measurements because emission lifetime response on external stimuli is independent of the probe concentration. Luminescent pH probes for microscopy are usually designed as fluorescence organic dyes [11,12,13] or phosphorescent organometallic coordination compounds [14,15], bearing pH-sensitive groups in their ligand environment. Organic fluorescent sensors appear to have poor photostability, small Stokes shift, low singlet lifetime, and weak lifetime response on pH variations (the best examples are SNARF-derivatives [16], 2.21 ns (pH 10)/0.49 ns (pH 5.5)). On the contrary, transition metal complexes usually show high photostability, long-lived emission with large Stokes shift. The latter two characteristics allow cutting off the sample autofluorescence, which helps to increase sensor sensitivity and the precision of the measurements [17]. The most studied phosphorescent sensors are platinum-group metal complexes, in particular cyclometalated Ir(III) compounds [18,19,20,21,22]. These emitters usually demonstrate high quantum yields and large lifetime values, easily modifiable excitation profile, and emission wavelength, which makes it possible to design the phosphorescent dyes with required photophysical characteristics. Flexible chemistry of ligand environment in the cyclometalated [Ir(**N^C**)_2_(**L^L**)]^n+^ complexes suggests a wide range of opportunities, including the introduction of pH-sensitive groups (NH_2_, COOH, pyridines, etc.) in different positions of the **N^C** [23,24,25] and auxiliary ligands [26,27,28,29,30,31,32,33]. This paves the way to obtaining luminescent pH sensors with versatile and tunable photophysical characteristics.

In this study, we focused on synthesis and investigation of the [Ir(**N^C**)_2_(**L^L**)] complexes bearing phenantroimidazole-based **N^C** ligand that contains nucleophilic nitrogen center potentially prone to protonation, thus giving a sensory response onto pH variations. Since insolubility in aqueous media is the main disadvantage of the majority of the typical cyclometalated iridium complexes suggested for pH-sensing in bioimaging [17], we also introduced -SO_3_Na and -COOH groups into the lateral **L^L**-ligand to increase hydrophilicity of the complex as a whole. The complexes obtained demonstrate phosphorescence in the yellow-orange region of the visible spectra. Photophysical characteristics of the complexes in solution vary upon addition of acid, showing a dramatic effect on the excited state lifetime values (τ) up to an order of magnitude. The τ vs. pH titration curves for **3** and **4** reveal a sigmoidal shape to give pK of 6.2 and 4.2, respectively, which allow carrying out measurements in the physiologically significant pH ranges. Living-cell experiments showed that complexes **3** and **4** internalize into cells, localize predominantly in lysosomes and late endosomes, and display relatively low toxicity at the complexes’ concentration sufficient for visualization and for obtaining a reliable lifetime map in the samples under study.

## 2. Results and Discussion

### 2.1. Synthesis of Ligand and Complexes

**Scheme 1:**
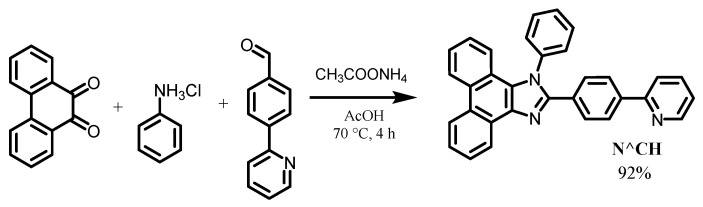
Synthesis of **N^CH**.

A new ligand precursor, 1-phenyl-2-(4-(pyridin-2-yl)phenyl)-1*H*-phenanthro[9,10-d]imidazole (**N^CH**), was readily obtained in good yield using Debus-Radziszewski reaction in a way reported to a closely analogous compound [34] (Scheme 1).

**Scheme 2:**
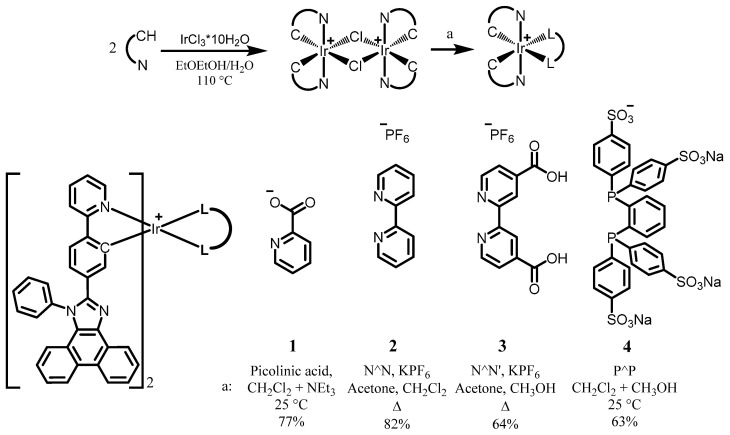
Synthesis of complexes **1**–**4**.

Using this ligand precursor and four chelating ligands (**L^L**) of different nature, we obtained a series of cationic and neutral [Ir(**N^C**)_2_(**L^L**)] complexes (**L^L** = picolinic acid (**1**), 2,2′-bipyridine (**2**), [2,2′-bipyridine]-4,4′-dicarboxylic acid (**3**), and sodium 4,4′,4″,4‴-(1,2-phenylenebis(phosphanetriyl))tetrabenzenesulfonate (**4**), Scheme 2). At the first stage, **N^C** coordination was performed starting from IrCl_3_ under commonly used conditions [35] to give the [Ir(**N^C**)_2_(μ-Cl)_2_Ir(**N^C**)_2_] dimer. Chloride ligands were then substituted with diimine or diphosphine ligands in neutral media or in the presence of triethylamine in the case of picolinic acid.

**Figure 1:**
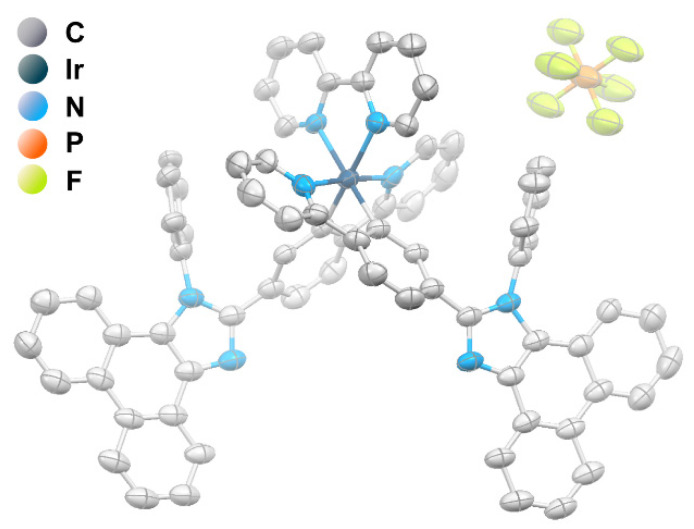
Perspective view of **2** in the solid state showing thermal ellipsoids at the 40% probability level. Hydrogen atoms are omitted for clarity.

**N^CH** and complexes **1**–**4** have been completely characterized by using NMR spectroscopy, ESI mass-spectrometry, and elemental analysis (for experimental details and spectral data (see Section 3 and Part 1 of Supporting Information file). The molecular structure of **2** in the solid state was established by XRD crystallography. (Figure 1, Table S1). It was found that the **N^C** ligand coordinates to iridium through adjacent pyridine and phenyl rings to form a five-membered metallacycle. This structural pattern is typical for Ir-cyclometalated compounds showing a distorted octahedron environment of the iridium ion with two nitrogen atoms of the **N^C** ligands in *trans*-position to each other, whereas carbon atoms and chelating atoms of the bidentate auxiliary ligands are disposed in the equatorial plane. The imidazole ring, thus, remains uncoordinated and accessible for protonation in acidic media.

The NMR spectroscopic data (1D ^1^H, ^1^H–^1^H COSY and NOESY, Figures S2–S7) obtained for **1**–**4** in solution are completely compatible with the coordination pattern found for complex **2** in the solid state (Figure 1). Relative intensity and multiplicity of resolved resonances, as well as their integral intensity in the 1D proton NMR spectra are in full agreement with the general structure of these compounds shown in Scheme 2 and Figure 1. In the ESI mass-spectra of the studied compounds (Figure S8) the dominating signals of the [M + H]^+^ cations were found for **N^CH** and complex **1**; complexes **2** and **3** displays the [M − PF_6_]^+^ signal, whereas the ESI^−^ mass-spectrum of complex **4** display a series of the signals corresponding to the negative ions generated by Na^+^ dissociation from the sulfo-group of the diphosphine ligand. The isotopic patterns of the signals in experimental spectra perfectly fit the calculated ones.

### 2.2. Photophysical Properties and Quantum Chemical Calculations for **N^CH** and Complexes ***1***–***4***

The **N^CH** precursor and **1**–**4** complexes exhibit effective emission in solution; their photophysical characteristics are summarized in Table 1 and Part 2 of SI; emission, excitation, and absorption spectra are presented in Figure 2, Figure 3 and Figures S9–S15.

**Figure 2:**
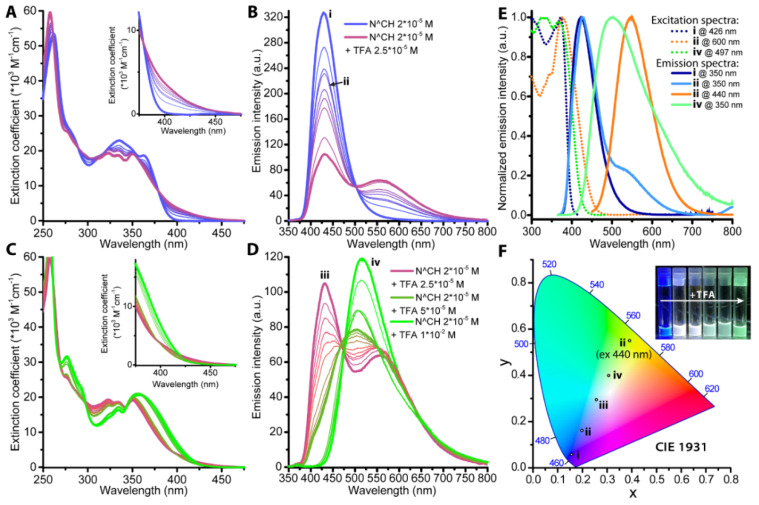
Spectral changes of **N^CH** in CH_2_Cl_2_ upon titration with TFA: (**A**,**C**) absorption spectra, (**B**,**D**) emission spectra λ_ex_ = 365 nm; (**E**) excitation and emission spectra of the representative solutions at different stages of the **N^CH** protonation chosen from the panels **B** and **D** (i—**N^CH**, ii—**N^CH** + TFA 10:1, iii—**N^CH** + TFA 1:1, iv—**N^CH** + TFA excess); and (**F**) CIE 1931 chromaticity diagram with an embedded photograph of the **N^CH** solution in CH_2_Cl_2_ at various TFA concentrations, λ_ex_ = 365 nm.

In the absence of acid, **N^CH** displays bright blue fluorescence (QY = 18%) with emission band maximum at 423 nm (Figure 2B) and lifetime in nanosecond domain. Absorption spectra of this chromophore (Figure 2A) display strong high energy (HE) absorption at ca 270 nm, which can be assigned to the π-π* transition (Table S4), whereas low energy (LE) absorption bands between 300 and 400 nm are related to the charge transfer transitions within this condensed aromatic system.

As expected, the addition of trifluoroacetic acid (TFA) to the dichloromethane (CH_2_Cl_2_) solution of **N^CH** affects both absorption and emission spectra leading to a gradual decrease in the intensity of the starting emission band and rise of a new one at ca. 550 nm (Figure 2B,D) to give a white color emission (Figure 2F, spectrum iii). A similar effect of generation of the white color emission was observed upon protonation of closely analogous push–pull azaheterocyclic systems [36,37]. At high TFA concentration, the emission spectrum displays strong bright-green band with the maximum at 510 nm (Figure 2D,F, spectrum iv). The emission and absorption spectra recorded upon titration of the **N^CH** solution with TFA at low acid concentration (up to ca. 1 equivalent of acid to one ligand) show clearly visible isosbestic points at 500 nm in emission (Figure 2B) and 289, 354, 380 nm in absorption (Figure 2A) spectra that indicate smooth conversion of the starting compound in a protonated form. Further addition of the acid (up to 5 × 10^−5^ M) gives a new set of isosbestic points at 470 nm in emission spectra (Figure 2D) and 296, 345, 395 nm in absorption spectra (Figure 2C), whereas in the concentrated TFA solutions (>1 mM) “isosbestisity” is not observed, probably due to several reactions taking place simultaneously. These observations allow hypothesizing successive protonation of **N^CH** at two available nucleophilic centers: pyridine and imidazole nitrogen atoms.

Monitoring of these ground-state transformations using ^1^H NMR spectroscopy with simultaneous control of emission spectra (Figure 4) made it possible to precisely assign the sequence of protonation reactions. At the first stage (low TFA concentration), the protonation occurs at the nitrogen atom of the pyridine ring because the protons of this aromatic system display the strongest low-field shift compared to the protons of the phenanthro-imidazole fragment. The spectrum at higher TFA concentration features considerable changes in the position and structure of the signals corresponding to protons of phenanthro-imidazole fragment where the protons at 1 and 4 carbon atoms even show additional coupling to the proton associated with imidazole nitrogen. It is also worth noting that the addition of the base (NEt_3_) neither affects emission nor NMR spectra, which is a clear indication of the electrophilic nature of reactive centers in **N^CH**.

According to the results of TD DFT calculations (see Part 3 in SI, Figure 5 and Figure S16, Tables S3 and S4), the observed emission occurs from the charge-transfer excited state with a considerable contribution of electron transfer from the phenanthro-imidazole fragment to the phenyl-pyridyl aromatic system (Figure 5). The shifts of emission wavelength in the course of successive protonation reactions are in complete agreement with the character of the emissive S_1_ excited state. In fact, the first stage of the protonation (occurs at pyridyl nitrogen) results in the reduction of the pyridine orbitals energy, accompanied by a decrease in the S_0_-S_1_ gap to give a red shift in the emission band. At the next stage, the protonation occurs at the imidazole nitrogen that in turn results in the reduction of the ground state energy of this fragment, increasing the energy gap between the orbitals taking part in the emissive transition.

**Figure 3:**
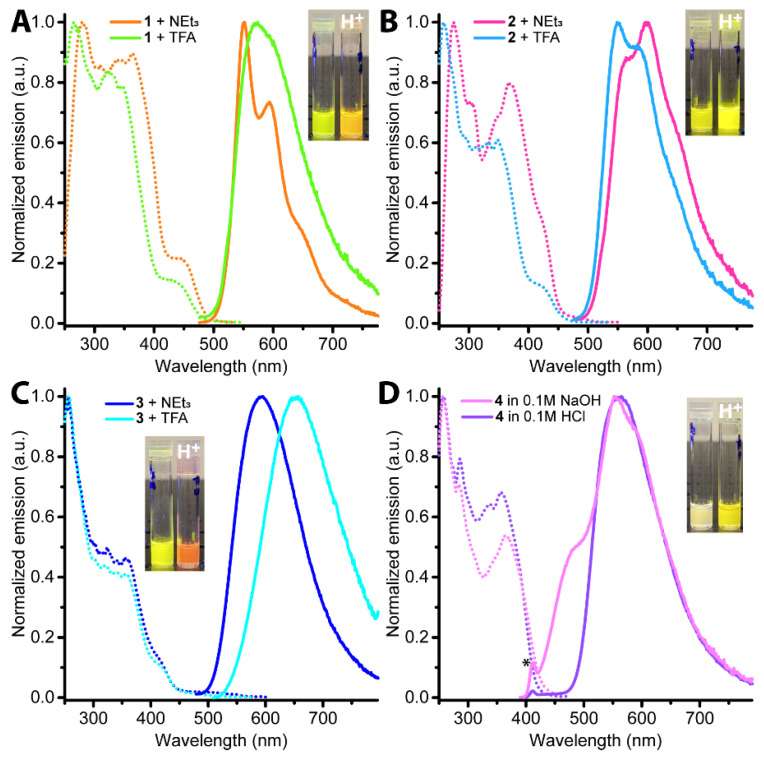
Normalized excitation (dashed line) and emission (solid line) spectra of **1**–**4** (**A**–**D** respectively). Emission spectra were recorded under excitation at 360 nm, excitation spectra were recorded at emission band maxima, dichloromethane, 1 × 10^−5^ M. A weak emission at 410 nm is generated by solvent (water) Raman resonance signal (*).

**Figure 4:**
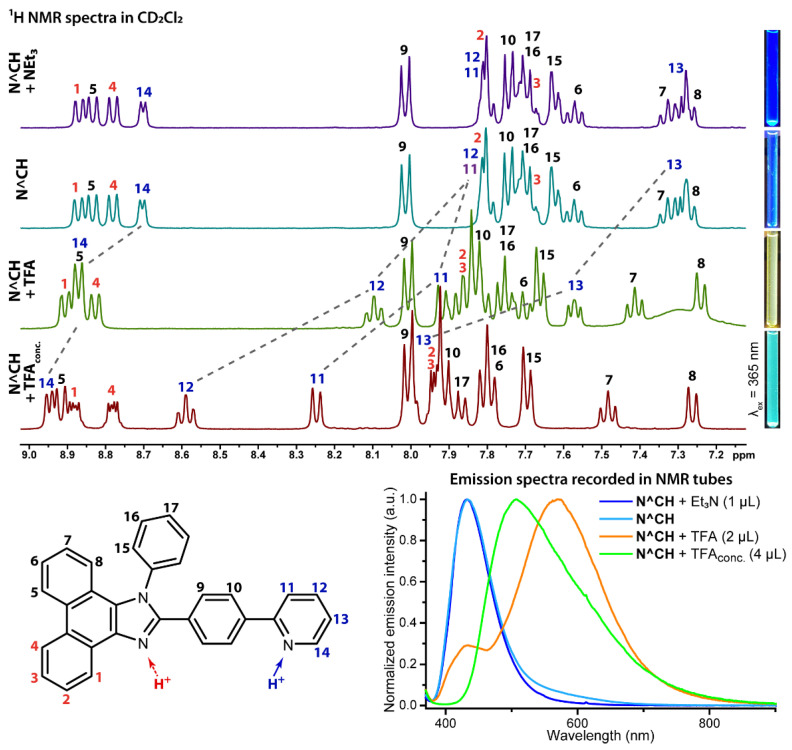
^1^H NMR spectra of **N^CH** upon addition of TFA. Bottom: **N^CH** emission spectra recorded in NMR tubes, λ_ex_ = 365 nm.

**Figure 5:**
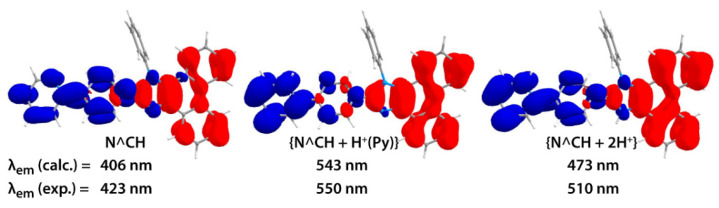
The decrease (blue) and increase (red) of electron density in the S_1_→S_0_ electronic transitions for **N^CH**, **{N^CH+H^+^(Py)}** containing protonated pyridine group, **{N^CH+2H^+^}** containing protonated pyridine and imidazole groups; calculated and experimental emission wavelengths are given in the bottom rows.

**Figure 6:**
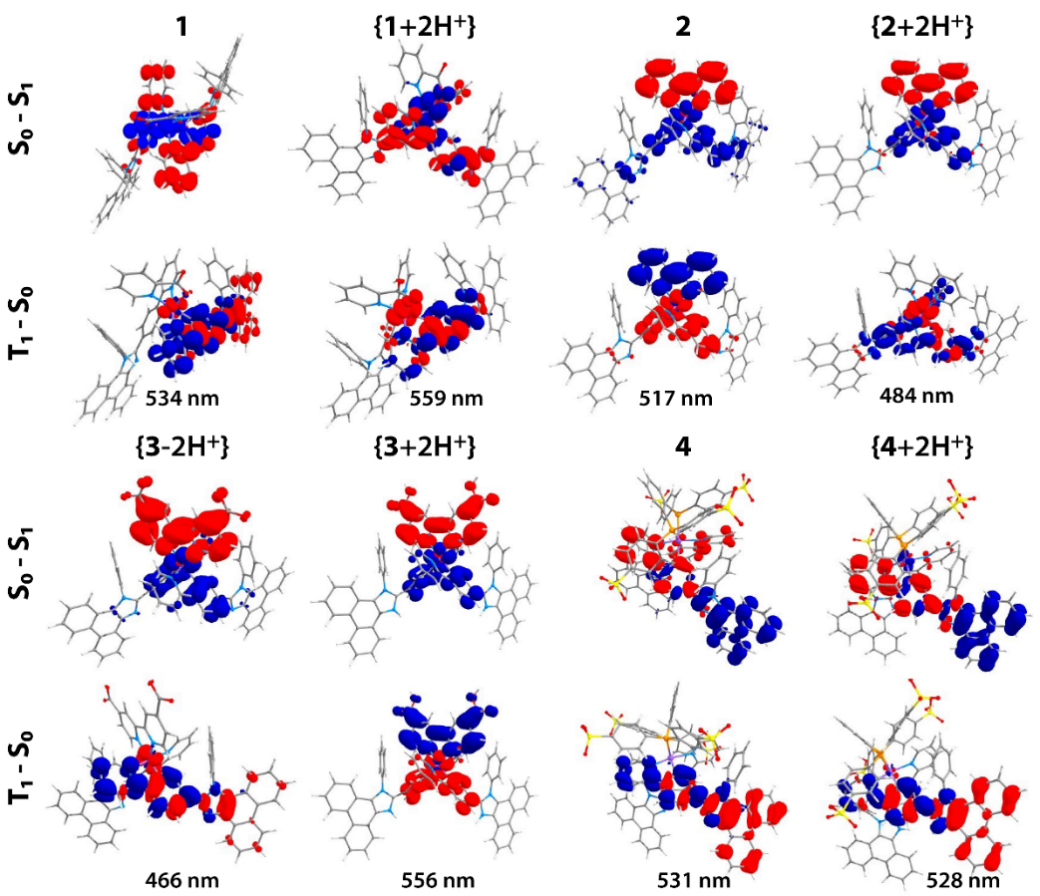
The decrease (blue) and increase (red) in electron density for S_0_**→**S_1_ and T_1_**→**S_0_ electronic transitions for complexes **1**–**4**, and calculated emission wavelengths. Note that {**3**−**2H^+^**} is the anion formed by the dissociation of two protons from the ligand carboxylic functions.

The complexes **1**–**4** display essentially similar absorption spectra with HE band at ca. 250–260 nm and LE absorption in the interval of 300–400 nm with a tail extending down to 450–460 nm (Figure 3 and Figure S9). However, the character of the lowest singlet excited states in these complexes vary considerably depending on the nature of the **L^L** ligand. According to the results of calculations for **1** (Figure 6 and Figure S18, Tables S5 and S6)**,** the major contribution into S_0_-S_1_ transition give metal to ligand (**N^C**) charge transfer (MLCT) and ligand (**N^C**) centered transition (LC) with negligible participation of the picolinate ligand orbitals. On the contrary, in **2** and **3** (deprotonated) the lowest energy S_0_-S_1_ excited states are of MLCT (**Ir→N^N**) and ligand to ligand (**N^C→N^N**) charge transfer (LLCT) character (Figure 6, Figures S20 and S22, Tables S9, S10, S14 and S15) that is evidently dictated by lower energy of the bipyridine π* orbitals compared to that of the phenanthro-imidazole fragment. The lowest energy transition in the complex **4** is predominantly of LC character with a small contribution of LLCT (**N^C→N^C**) without participation of the metal and diphosphine orbitals in this transition (Figure 6 and Figure S24, Tables S18 and S19).

All complexes are luminescent in solution with emission band maxima ranging from 500 to 650 nm (Figure 3). Large Stokes shift and lifetime in microsecond domain point to the triplet nature of these emissive excited states, i.e., phosphorescence (Table 1). Complex **1** demonstrates well-resolved emission band with the major component maximum at 553 nm and vibronic progression of ca. 1300 cm^−1^, see Figure 3 and Table 1. This observation is in complete agreement with the assignment of emissive triplet state to ^3^LC character (Table S6, Figure 6). Upon protonation, the emission band demonstrates bathochromic shift accompanied by the disappearance of the band’s fine-structure. These observations can be assigned to a considerable increase of the ^3^MLCT (**Ir→N^C**) contribution into the emissive T_1_-S_0_ transition in addition to the ^3^LC character and to the reduction of the **N^C** orbitals energy upon the ligand protonation (Table S8, Figure 6). Complex **2** shows emission from the excited state of the mixed charge transfer character, ^3^LLCT(**N^N→N^C)/**^3^MLCT(**N^N→Ir)** (Table S10, Figure 6). Protonation of **2**, which evidently takes place at the **N^C** ligand, gives a weak hypsochromic shift of emission band (ca. 20 nm) and results in essential alteration of the T_1_ character. The emission of the protonated form occurs from the mixed ^3^LC(**N^C)/**^3^MLCT(**N^C→Ir)** excited state (Table S12, Figure 6). In contrast to **2**, the complex **3** demonstrates an opposite (bathochromic) emission band shift upon protonation. The starting complex emits from ^3^LC (**N^**C)/^3^MLCT(**N^C→Ir)** excited state at 600 nm, whereas the emission band is shifted to 650 nm upon protonation and displays ^3^LLCT(**N^N→N^C**)/^3^MLCT(**N^C→Ir)** character according to the results of calculations (Tables S15 and S17, Figure 6). The complex **4** in both protonated and deprotonated forms shows a broad emission band at ca. 560 nm, which may be assigned to the intraligand (**N^C**) charge transfer ^3^ILCT transition (Tables S19 and S21, Figure 6). A shoulder at 480 nm displays nanosecond lifetime (Table 1) and independence on oxygen concentration (cf. spectra in Figure 3 and Figure S14) that indicates its origin from singlet excited state. A similar competitive singlet and triplet emissions were observed in orthometalated platinum diphosphine complexes [38] where competition between intersystem crossing and internal conversion also results in a deviation from Kasha−Vavilov’s rule.

**Figure 7:**
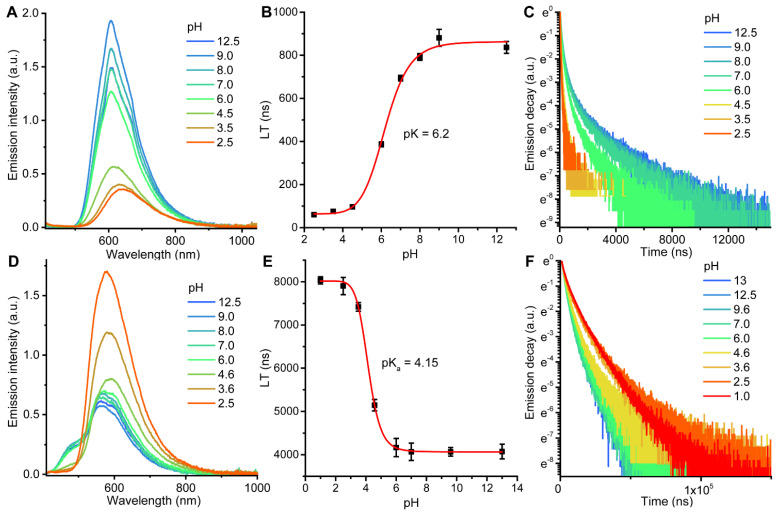
Dependence of emission intensity (**A**,**D**), lifetime of the excited state (**B**,**E**), and normalized emission decay curves (**C**,**F**) of **3** (**A**–**C**, λ_ex_ = 355 nm, λ_em_ = 600 nm) and **4** (**D**–**F**, λ_ex_ = 355 nm, λ_em_ = 560 nm) upon pH variations in aerated aqueous buffer solution (pH 12.5, 8.0, 7.0, 6.0, 2.5—phosphate buffer solution, pH 4.75, 4.5, and 3.5—citrate buffer solution, pH 9.0 and 9.6—borate buffer solution, for **3** buffer/acetone 9/1; *v/v*), 293K. Lifetime (LT) of the probes were calculated from bi- (for **4**) and tri- (for **3**) exponential decay fit as intensity-weighted average lifetime (τ_av_, see Equation (1)).

Photophysical properties (emission spectra and lifetime of excited state) of **3** and **4** were also studied in aerated aqueous buffer solutions (Figure 7). For **3**, variations of emission intensity and excited-state lifetime on pH were studied in the solutions containing *v/v* 10% of acetone, the latter was added to dissolve **3** in aqueous media. The phosphorescent band expectedly displays a bathochromic shift upon solution acidification accompanied by a substantial decrease in emission intensity and lifetime. The decay of the excited state can be described as a tri-exponential decay and the data shown in Figure 7C represent the averaged (τ_av_, see Part 3, Equation (1)) values of these three lifetimes. This complex behavior most probably indicates the emission from various protonated forms of the complex. The lifetime dependence on pH displays a typical sigmoidal curve with the pK magnitude of ca. 6.2 that matches well physiologically relevant interval from 4.0 to 8.5 to give more than an order of magnitude increase in the lifetime values in this pH range. Complex **4** is water-soluble, thus, the measurements were carried out in the solutions without organic solvent. The emission of **4** increases considerably upon acidification. Emission decay can be fit with bi-exponential function giving average lifetime values of ca. 4 µs at pH > 6 and ca. 8 µs at pH < 3. A substantial change of the lifetime is observed in the pH interval from 2.5 to 6; the sigmoidal fit of the data gives pK value of 4.15. These observations indicate that both complexes are potentially suitable for application as pH sensors in biological systems by using phosphorescence lifetime imaging (PLIM) mode of luminescent microscopy. Therefore, we performed several experiments to test the applicability of these compounds for cellular staining and pH sensing in living cells.

### 2.3. Living Cell Imaging Using ***1***–***4***

**Figure 8:**
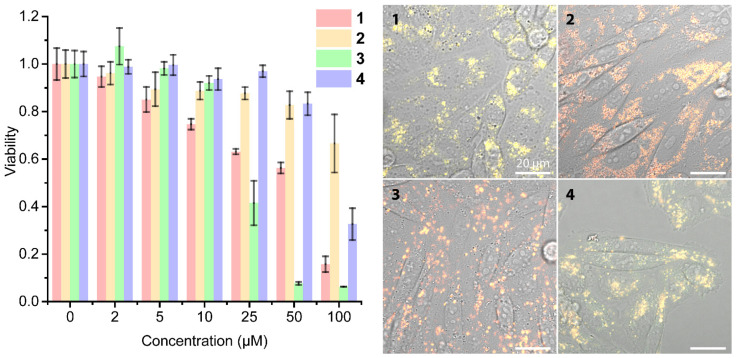
**Left**: MTT assay of CHO-K1 cells after incubation with complexes **1**–**4** for 24 h at different concentrations. Cell viability of control cells (without probe) was taken for 1. The data are shown as mean ± standard deviation. N = 6 repetitions for CHO-K1 cells. **Right**: Confocal microscopy of CHO-K1 cells incubated with complexes **1**–**3** (5 µM, 24 h) and **4** (25 µM, 24 h). Green, red, and DIC (differential interference contrast) channels are merged. Scale bar 20 µm.

We carried out experiments on living Chinese Hamster ovary cells (CHO-K1), to evaluate the toxicity and intercellular localization of **1**–**4**. The cellular toxicity of these probes was evaluated by using MTT colorimetric assay (Figure 8, left). It was found that after 24 h incubation, the complexes demonstrate low cellular toxicity at concentrations up to 10 µM and can be used for living cells staining below this concentration limit. Confocal microscopy of CHO-K1 cells incubated with complexes **1**–**4** for 24 h is shown in Figure 8, right. The compounds **1**–**3** at a concentration of 5 µM easily internalize into cells showing localization in some intracellular compartments. Complex **4** demonstrates considerably lower cellular uptake, very probably due to the negative charge of the molecular ion due to the acidic character of the diphosphine, that made us to use a significantly higher concentration (25 µM), which is still lower than the toxicity limit for this probe, see Figure 8.

**Figure 9:**
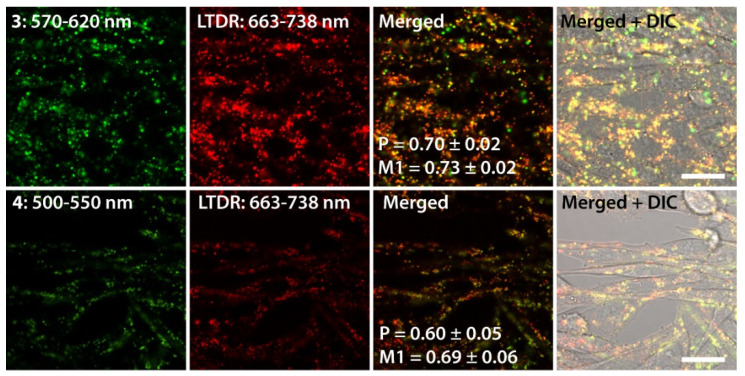
Co-staining of CHO-K1 cells with complexes **3** (5 µM, 24 h) and **4** (25 µM, 24 h) and Lysotracker Deep Red (50 nM, 30 min). Pearson’s (P) and Mander’s (M1) coefficients are given in the merged picture.

Intracellular localization of **3** and **4**, which showed prospect for application as pH probes, was studied in detail by co-staining with endolysosomes selective dye (Lysotracker Deep Red, LTDR), see Figure 9. For **3**, co-localization coefficients of ca. 0.7 suggests predominating endosomal-lysosomal localization of the complex. However, for **4**, the co-localization with LTDR is lower indicating its partial localization in some other compartments or in cytosol.

**Figure 10:**
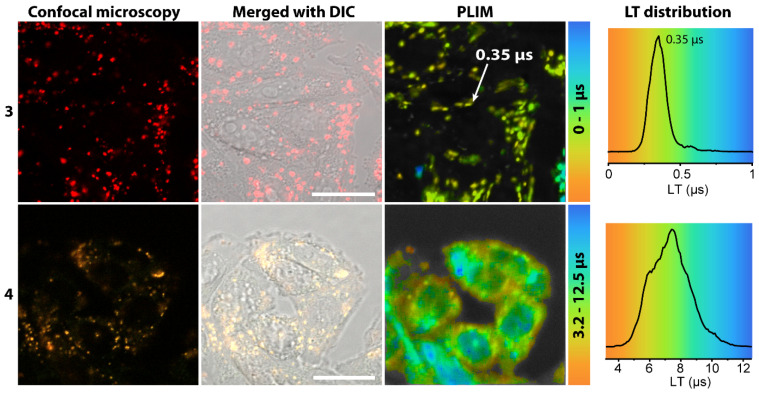
Confocal and PLIM-images of CHO-K1 cells incubated with complex **3** (Top, 5 µM, 24 h) and **4** (Bottom, 25 µM, 24 h). **Left**: confocal image and merged confocal image and DIC. **Central:** PLIM image. **Right**: lifetime distribution for the PLIM image. Excitation 405 nm, 37 °C and 5% CO_2_, normoxia.

Using PLIM, we assessed the phosphorescence lifetime of **3** and **4** inside living cells (Figure 10). PLIM imaging of the cells incubated with **3** indicates that the probe’s PLIM signal is localized in the same areas where phosphorescence was detected by confocal microscopy. The PLIM data were processed as tri-exponential phosphorescence decay (χ^2^ ≤ 1.2) similar to the data treatment in solution, *vide supra*. The intensity-weighted average lifetime values (τ_av_, see Part 3, Equation (1)) found in the cells fall in the lifetime range found for **3** in aerated buffer solutions at pH 5.0 (see Figure 7). These findings are compatible with the localization of the probe in lysosomes, pH of which varies in the range 4.5–6.0. Complex **4** displays weak emission in luminescent confocal microscopy and in PLIM. The phosphorescent signal is detectable not only from compact vesicles, but evenly spread throughout the whole cell. PLIM data were fitted in bi-exponential mode, similar to the data treatment in solution. Lifetime (τ_av_) varies in the broad range from 5 to 10 µs with an average value of ca. 7.5 µs that is evidently a result of even distribution of **4** in the cell compartments of different nature.

The preliminary cellular imaging experiments with complexes **3** and **4** showed their internalization into cells and predominant localization in lysosomes. Lifetimes found in the PLIM cell experiments and recalculated to the pH values using the calibration curves obtained in aqueous buffer solutions fit well the physiological range, particularly taking into account the probes localization. However, the further optimization of the emitter’s properties should be done to use these compounds as quantitative pH sensors in imaging experiments. (a) The complexes demonstrate minor (**3**) to high (**4**) sensitivity to oxygen concentration. Therefore, it is necessary either to minimize the probe lifetime sensitivity to O_2_ (by the chromophore shielding with bulky ligands, its encapsulation into polymeric nanoparticles, etc. [17]) or to use the probe simultaneously with an oxygen sensor to correct the lifetime data for the oxygen quenching effect; (b) It is also necessary to evaluate and minimize the other distorting effects of such factors as media viscosity, interaction with biomolecules and metal ions; (c) The processing of multiexponential decay curves requires high emission intensity to obtain reliable statistics of the decay photons and high precision lifetime data. Thus, increase in the sensor emission quantum yield is also highly desirable.

## 3. Materials and Methods

### 3.1. Synthesis of the Ligand and Complexes

General comments. Cyclometalated iridium dimer [(**N^C**)_2_IrCl]_2_ were prepared according to the conventional procedure [35,39,40] by the reaction of IrCl_3_ × 10H_2_O with the corresponding **N^CH** ligand precursor in 2-ethoxyethanol/water mixture under heating at 110 °C. The solid obtained was collected, washed with water and methanol, and used in the following reactions without additional purification. Phenanthrene-9,10-dione, aniline hydrochloride, 4-(pyridin-2-yl)benzaldehyde, picolinic acid, and [2,2′-bipyridine]-4,4′-dicarboxylic acid were purchased from Sigma Aldrich (Merck, Munich, Germany) and used as received. 1,2-Bis(di-4sulfonatophenylphosphino)benzene tetrasodium salt DMSO adduct was obtained from Strem Chemicals.

The solution ^1^H, ^31^P{^1^H} NMR and ^1^H-^1^H COSY spectra were recorded on a Bruker Avance 400 spectrometer (Bruker, Germany) with chemical shifts referenced to residual solvent resonances. Electrospray ionization (ESI) mass spectra were recorded using a maXis II ESI–QTOF instrument (Bruker, Germany) in the ESI^+^ and ESI^−^ mode. Microanalyses were carried out at the analytical laboratory of the University of Eastern Finland using a vario MICRO cube CHNS-analyzer (Elementar, Langenselbold, Germany).

1-Phenyl-2-(4-(pyridin-2-yl)phenyl)-1*H*-phenanthro[9,10-d]imidazole **(N^CH)**. A mixture of phenanthrene-9,10-dione (700 mg, 3.395 mmol), aniline hydrochloride (1100 mg, 8.487 mmol), 4-(pyridin-2-yl)benzaldehyde (622 mg, 3.395 mmol), and ammonium acetate (654 mg, 8.487 mmol) was stirred in 20 mL of glacial acetic acid at 70 °C for 4 h. During the reaction, orange colour of the reaction mixture changed to dark green. The target compound was formed as a white precipitate with green emission under UV irradiation at 365 nm. After 4 h, the volume of the solvent was reduced to 5 mL using rotary evaporator and heating. After cooling to room temperature, the precipitate was separated using centrifugation and washed with methanol (3 × 5 mL) and diethyl ether (3 × 5 mL). The product was recrystallized from dichloromethane and dried under vacuum. Yield: 1398 mg (3.124 mmol, Mw = 447.54 g/mol), 92%. ^1^H NMR (400 MHz, CDCl_3_, 298 K): δ 8.91 (dd, ^3^*J_H-H_* = 8.0, ^4^*J_H-H_* = 1.2 Hz, 1H), 8.79 (d, ^3^*J_H-H_* = 8.3 Hz, 1H), 8.73 (d, ^3^*J_H-H_* = 8.3 Hz, 1H), 8.69 (d, ^3^*J_H-H_* = 4.7 Hz, 1H), 7.95 (d, ^3^*J_H-H_* = 8.5 Hz, 2H), 7.76 (td, ^3^*J_H-H_* = 7.8, ^4^*J_H-H_* = 1.1 Hz, 1H), 7.76–7.70 (m, 4H), 7.67 (td, ^3^*J_H-H_* = 7.8, ^4^*J_H-H_* = 1.5 Hz, 1H), 7.65–7.59 (m, 3H), 7.56 (dd, ^3^*J_H-H_* = 8.0, ^4^*J_H-H_* = 1.8 Hz, 2H), 7.53 (td, ^3^*J_H-H_* = 8.3, ^4^*J_H-H_* = 1.4 Hz, 1H), 7.30–7.21 (m, 3H) ppm. ES MS (*m/z*): [M+H]^+^ 448.1764 (calc. 448.1814). Anal. calc. for C_32_H_21_N_3_*1/2H_2_O (%): C 84.19; H 4.86; N 9.20. Found: C 83.76; H 4.74; N 9.25.

[(**N^C**)_2_IrCl]_2_. **N^CH** (100 mg, 0.222 mmol) was heated in a round-bottom flask in 13 mL of 2-etoxyethanole at 60 °C. When the organic ligand was completely dissolved, the solution of IrCl_3_ × 10H_2_O (53 mg, 0.112 mmol) in 5 mL of water was added to the flask. The reaction mixture was purged with argon for 15 min and refluxed for 4 days at 110 °C. During heating, the mixture turned red, and dark-red precipitate was formed. Blue emission of **N^CH** under UV light (365 nm) gradually disappeared. The reaction mixture was then evaporated to dryness, the precipitate was washed with methanol (2 × 10 mL) and diethyl ether (4 × 10 mL) and dried under vacuum. The red solid obtained is non-soluble in the majority of solvents except DMSO, in which the decomposition of dimer compound and coordination of DMSO are possible. Thus, the solids obtained were used without additional purification in the further syntheses. Yield of the iridium dimer: 120 mg (0.053 mmol, Mw = 2245.50 g/mol), 98%.

Complex **1.** [(**N^C**)_2_IrCl]_2_ (30 mg, 0.013 mmol) and picolinic acid (4 mg, 0.032 mmol) were added to 10 mL of dichloromethane. One drop of NEt_3_ (ca. 50 µL) was then added under vigorous stirring. Upon base addition, the iridium dimer was dissolved and the solution colour turned yellow. The reaction mixture was stirred at room temperature for 1 h, followed by solvent evaporation under vacuum. The solid precipitate was washed with diethyl ether, dissolved in CH_2_Cl_2_ and passed through silica layer using CH_2_Cl_2_ + MeOH 4:1 as eluent. The product was recrystallized from CH_2_Cl_2_/hexane mixture by slow evaporation of CH_2_Cl_2_ from the solution at RT. Yield: 24 mg of yellow-orange crystalline compound (0.020 mmol, 77%). ^1^H NMR (400 MHz, DMSO-*d*_6_, 298 K): δ 8.90 (d, ^3^*J_H-H_* = 8.4 Hz, 1H), 8.85 (d, ^3^*J_H-H_* = 8.4 Hz, 1H), 8.55 (td, ^3^*J_H-H_* = 8.7 Hz, 2H), 8.45 (d, ^3^*J_H-H_* = 5.4 Hz, 1H), 8.29 (d, ^3^*J_H-H_* = 8.0 Hz, 1H), 8.22 (d, ^3^*J_H-H_* = 8.4 Hz, 1H), 8.15–8.08 (m, 2H), 8.01–7.94 (m, 2H), 7.93 (d, ^3^*J_H-H_* = 8.2 Hz, 1H), 7.79–7.73 (m, 3H), 7.70–7.47 (m, 13H), 7.41–7.34 (m, 5H), 7.33–7.26 (m, 5H), 7.23 (d, ^3^*J_H-H_* = 5.2 Hz, 1H), 7.05 (d, ^3^*J_H-H_* = 8.3 Hz, 1H), 7.00–6.86 (m, 2H), 6.53 (d, ^4^*J_H-H_* = 1.6 Hz, 1H), 6.35 (d, ^4^*J_H-H_* = 1.3 Hz, 1H) ppm. ES MS (*m/z*): [M+2H]^2+^ 604.6661 (calc. 604.6669). Anal. calc. for C_70_H_44_IrN_7_O_2_*CH_2_Cl_2_ (%): C 65.99; H 3.59; N 7.59. Found: C 65.40; H 3.90; N 7.48.

Complex **2.** [(**N^C**)_2_IrCl]_2_ (30 mg, 0.013 mmol), 2-2′bypiridine (5 mg, 0.032 mmol), and potassium hexafluorophosphate (7 mg, 0.038 mmol) were refluxed in 20 mL of acetone for 24 h. The resulting yellow solution was dried under vacuum, the precipitate was washed with diethyl ether, dissolved in CH_2_Cl_2_ and passed through silica layer using CH_2_Cl_2_ + MeOH 4:1 as eluent. The product was recrystallized from CH_2_Cl_2_/hexane mixture via slow evaporation of CH_2_Cl_2_ from the solution at RT. Yield: 30 mg of yellow crystalline compound (0.022 mmol, 82%). ^1^H NMR (400 MHz, DMSO-*d*_6_, 298 K): δ 8.90 (m, ^3^*J_H-H_* = 8.5, 8.8 Hz, 2H), 8.86 (d, ^3^*J_H-H_* = 8.5 Hz, 1H), 8.56 (dd, ^3^*J_H-H_* = 8.1, ^4^*J_H-H_* = 1.0 Hz, 1H), 8.32 (td, ^3^*J_H-H_* = 8.1, ^4^*J_H-H_* = 1.3 Hz, 1H), 8.27 (d, ^3^*J_H-H_* = 8.4 Hz, 1H), 7.99–7.93 (m, 2H), 7.77 (td, ^3^*J_H-H_* = 7.2, ^4^*J_H-H_* = 0.7 Hz, 1H), 7.72–7.63 (m, 3H), 7.61–7.52 (m, 3H), 7.46 (dd, ^3^*J_H-H_* = 5.5, ^4^*J_H-H_* = 0.7 Hz, 1H), 7.39–7.27 (m, 6H), 7.05 (d, ^3^*J_H-H_* = 7.9 Hz, 1H), 6.34 (d, ^4^*J_H-H_* = 1.6 Hz, 1H) ppm. ES MS (*m/z*): [M-PF_6_+H]^2+^ 621.1845 (calc. 621.1853).

Complex **3.** [(**N^C**)_2_IrCl]_2_ (30 mg, 0.013 mmol), [2,2′-bipyridine]-4,4′-dicarboxylic acid (7 mg, 0.029 mmol), and potassium hexafluorophosphate (7 mg, 0.038 mmol) were refluxed in the mixture of acetone (10 mL) and methanol (5 mL) overnight. The resulting orange reaction mixture was dried under vacuum, the precipitate was dissolved in MeOH/CH_2_Cl_2_ mixture and passed through silica layer. The product was recrystallized from MeOH/CH_2_Cl_2_ mixture via slow diffusion of diethyl ether in the solution at RT. Yield: 25 mg of yellow crystalline compound (0.017 mmol, 64%). ^1^H NMR (400 MHz, DMSO-*d*_6_, 298 K): δ 9.16 (s, 1H), 8.90 (d, ^3^*J_H-H_* = 9.0 Hz, 1H), 8.85 (d, ^3^*J_H-H_* = 9.0 Hz, 1H), 8.56 (dd, ^3^*J_H-H_* = 8.0, ^4^*J_H-H_* = 1.1 Hz, 1H), 8.27 (d, ^3^*J_H-H_* = 8.6 Hz, 1H), 7.99 (d, ^3^*J_H-H_* = 5.1 Hz, 1H), 7.96 (t, ^3^*J_H-H_* = 8.3 Hz, 1H), 7.94 (d, ^3^*J_H-H_* = 8.3 Hz, 1H), 7.77 (t, ^3^*J_H-H_* = 7.7 Hz, 1H), 7.68 (t, ^3^*J_H-H_* = 7.6 Hz, 2H), 7.56–7.51 (m, 4H), 7.42 (dd, ^3^*J_H-H_* = 5.9, ^4^*J_H-H_* = 0.8 Hz, 1H), 7.36 (d, ^3^*J_H-H_* = 7.9 Hz, 1H), 7.33–7.27(m, 4H), 7.05 (dd, ^3^*J_H-H_* = 8.3, ^4^*J_H-H_* = 0.7 Hz, 1H), 6.34 (d, ^3^*J_H-H_* = 1.6 Hz, 1H) ppm. ES MS (*m/z*): [M-PF_6_]^+^ 1329.3658 (calc. 1329.3436).

Complex **4.** [(**N^**C)_2_IrCl]_2_ (75 mg, 0.033 mmol) was added to 20 mL of deoxygenated dichloromethane, 1,2-bis(di-4sulfonatophenylphosphino)benzene tetrasodium salt DMSO adduct (65 mg, 0.076 mmol) was dissolved in 20 mL of deoxygenated CH_3_OH. The obtained solution was then dried under vacuum. The resulting solid was dissolved in CH_3_OH/CH_2_Cl_2_ mixture, passed through Celites layer and precipitated using diethyl ether. The amorphous solid was washed with diethyl ether (3 × 10 mL), and small portions of methanol (2 × ca. 1 mL) to remove the excess of diphosphine. The final product was recrystallized from CH_3_OH/CH_2_Cl_2_ mixture via evaporation of the solvents at RT. Yield: 80 mg of pale-yellow powder (0.042 mmol, 63%). ^1^H NMR (400 MHz, DMSO-*d*_6_, 298 K): δ 8.30–7.98 (m, 2H), 7.80 (d, ^3^*J_H-H_* = 8.3 Hz, 1H), 7.43 (s, 1H), 7.29 (s, 1H), 7.18 (t, ^3^*J_H-H_* = 9.3 Hz, 4H), 7.08 (d, ^3^*J_H-H_* = 5.0 Hz, 3H), 7.01–6.86 (m, 7H), 6.79 (m, 2H), 6.73 (d, ^3^*J_H-H_* = 6.0 Hz, 1H), 6.59 (m, 3H), 6.39 (d, ^3^*J_H-H_* = 7.7 Hz, 2H), 6.22 (d, ^3^*J_H-H_* = 8.6 Hz, 1H), 5.85 (s, 1H), 5.80 (t, ^3^*J_H-H_* = 6.6 Hz, 1H), 5.51 (t, ^3^*J_H-H_* = 8.8 Hz, 2H) ppm. ^31^P NMR (162 MHz, DMSO-*d*_6_, 298 K): δ 19.59 (s) ppm. ^1^H NMR (400 MHz, CD_3_OD, 298 K): δ 8.92–8.88 (m, ^3^*J_H-H_* = 8.4 Hz, 2H), 8.54–8.52 (m, 1H), 8.22 (m, 1H), 8.09 (s, 1H), 8.00–7.91 (m, 6H), 7.84–7.82 (m, 2H), 7.77–7.65 (m, 6H), 7.58 (t, *J* = 8.7 Hz, 5H), 7.48 (d, ^3^*J_H-H_* = 5.6 Hz, 1H), 7.35 (m, 3H), 7.21 (d, ^3^*J_H-H_* = 7.7, ^4^*J_H-H_* = 1.6 Hz, 2H), 7.02 (d, ^3^*J_H-H_* = 8.0 Hz, 1H), 6.58 (s, 2H), 6.35 (t, *J* = 8.9 Hz, 2H) ppm. ^31^P NMR (162 MHz, CD_3_OD, 298 K): δ 20.54 (s) ppm. ES MS (*m/z*): [M-3Na]^3+^ 615.7417 (calc. 615.7415).

### 3.2. X-ray Diffraction Analysis

The crystal of complex **2**, suitable for XRD analysis, was grown in CH_2_Cl_2_/hexane mixture via slow evaporation of CH_2_Cl_2_ from the solution at RT. The compound crystallizes in different forms, however, only one type of crystal was suitable for single-crystal analysis. The crystal structure was determined by the means of single-crystal XRD analysis using a XtaLAB Synergy HyPix diffractometer with monochromated Cu*Kα* radiation for the data collection at a temperature of 100 K. Diffraction data were processed in the CrysAlisPro program [41]. Using Olex2 [42], the structure was solved with the SHELXS [43] structure solution program using direct methods and refined with the SHELXL [44] refinement package using least squares minimization. The unit cells of **2** contain disordered solvent molecules which have been treated as a diffuse contribution to the overall scattering without specific atom positions by SQUEEZE/PLATON [45]. The total potential solvent accessible Void Vol in **2** is 1764 Å^3^ and electron Count Voids/Cell = 388 that is approximately equal to two hexane molecule per formula unit. It was found that the unit cell contains 1.5 PF_6_ species per one [(**N^C**)_2_Ir(N^N)] molecule. This may indicate that half of the complex molecules in the crystal cell are protonated, very probably at the imidazole ring of the **N^C**-ligand. Crystal Data for C_74_H_48_IrN_8_ (PF_6_)_1.5_ (*M* = 1458.86 g/mol): monoclinic, space group P2_1_/c (no. 14), *a* = 23.7979(4) Å, *b* = 13.6503(2) Å, *c* = 24.1503(5) Å, *α = γ = 90*°, *β* = 115.832(2)°, *V* = 7061.2(3) Å^3^, *Z* = 4, *T* = 99.8(9) K, μ(Cu Kα) = 4.575 mm^−1^, *D_calc_* = 1.372 g/cm^3^, 56,996 reflections measured (7.344° ≤ 2Θ ≤ 160.204°), 14,887 unique (*R*_int_ = 0.0472, R_sigma_ = 0.0395) which were used in all calculations. The final *R*_1_ was 0.0559 (I > 2σ(I)) and *wR*_2_ was 0.1718 (all data). Crystal size 0.26 × 0.2 × 0.12 mm^3^. Supplementary crystallographic data for this paper have been deposited at Cambridge Crystallographic Data Centre (CCDC 2127882) and can be obtained free of charge via www.ccdc.cam.ac.uk/structures/ (accessed on 28 December 2021).

### 3.3. Photophysical Measurements

Photophysical measurements in solution were carried out using distilled CH_2_Cl_2_, methanol, water, and aqueous buffer solutions (pH 12.5, 8.0, 7.0, 6.0, 2.5 – phosphate buffer saline 10 mM, pH 4.8–3.5–sodium citrate buffer solution 10 mM, pH 9.0 and 9.6 – sodium borate buffer solution 50 mM). Deoxygenation of the solutions for lifetime and quantum yield measurements was performed by purging of N_2_ through the solutions for 20–30 min. UV/Vis spectra were recorded with a Shimadzu UV-1800 spectrophotometer at concentrations of ca. 1 × 10^−5^ M in 1 cm quartz cuvettes. Emission spectra were measured using an Avantes AvaSpec-2048 × 64 spectrometer (Avantes, Apeldoorn, The Netherlands). Excitation spectra in solution and some emission spectra were recorded on a FluoMax-4 (JY Horiba Inc., Japan) spectrofluorimeter at concentrations of ca. 1 × 10^−5^ M. The emission quantum yields were determined by the comparative method [46] using Ru(bpy)_3_^+^ in aerated water (Φ_r_ = 0.040) [47] as the reference with the refractive indices of CH_2_Cl_2_, water, and methanol equal to 1.42, 1.33, and 1.328, respectively. Phosphorescence lifetime measurements were carried out by using a device comprised of a Pulse laser TECH-263 Basic (wavelength 355 nm, pulse width 5 ns, repetition frequency 1000–500 Hz) (Laser Export, Moscow, Russia), a Hamamatsu H10682-01 photon-counting head (Hamamatsu, Hamamatsu, Japan), a FASTComTec MCS6A1T4 multiple-event time digitizer (FAST ComTec, Oberhaching, Germany), and an Ocean Optics monochromator Monoscan-2000 (interval of wavelengths 1 nm; Ocean Optics, Largo, FL, USA). Temperature control was performed by using a Quantum Northwest qpod-2e (Quantum Northwest Inc., Liberty Lake, WA, USA) cuvette sample compartment. Fluorescence lifetime was recorded on a HORIBA Scientific FluoroLog-3 spectrofluorometer (JY Horiba Inc., Kyoto, Japan). The lifetime data were fit using the Jobin-Yvon software package and the Origin 9.0 program. The intensity-weighted average lifetimes for bi- and tri-exponential decay were calculated using the equations:

(1) $$\tau_{av}=\frac{A_{1}\tau_{1}^{2}+A_{2}\tau_{2}^{2}}{A_{1}\tau_{1}+A_{2}\tau_{2}},\tau_{av}=\frac{A_{1}\tau_{1}^{2}+A_{2}\tau_{2}^{2}+A_{3}\tau_{3}^{2}}{A_{1}\tau_{1}+A_{2}\tau_{2}+A_{3}\tau_{3}},$$

where *A_i_* is the weight of the i-exponent and τ*_i_* is the corresponding lifetime component.

### 3.4. Computational Details

The fully optimized structures of ground and excited triplet states were obtained within the DFT for all compounds under consideration. The calculations were realized using the Gaussian-16 program [48]. The Austin–Frisch–Petersson functional with dispersion (APFD) [49] was chosen for the most accurate description of experimental trends. The Stuttgart-Dresden effective core pseudopotential and the corresponding basis set were used for iridium [50]. The Pople’s 6-31G* Gaussian-type function basis set was chosen for carbon and hydrogen atoms, for all other atoms, 6-311+G* basis set was used [51]. The non-specific solvation effects of appropriate solvents were taken into account by the polarizable continuum model (PCM) [52].

Emission energies were obtained as the difference between energies of optimized triplet and singlet states. Fluorescence wavelengths of the precursor and its protonated forms were calculated in the TD-DFT methodology. The electronic absorption spectra were calculated within TD-DFT with 200 excited states for all complexes and with 150 states for **N^CH**. The convoluting of UV/Vis spectra from calculated oscillator strengths were obtained using the method described in ref. [53] modified for Lorentzian broadening. All the calculated spectra were compared with the experimental ones.

Two approaches were used to describe the displacement of the electron density during absorption and emission transitions and to determine their character. A qualitative picture was established by the construction of natural transition orbitals (NTO) [54]. A number of electrons transferred between the discussed parts of the molecules have been obtained by IFCT (Interfragment charge transfer) method [55]. The Multiwfn 3.6 program [55] the program was used for both methods. The changes in electronic density *Δρ* during the S_0_→S*_i_* transitions were calculated as:

(2) $$\Delta \rho \left(S_{0}\to S_{i}\right)=\underset{k}{\sum }{\left|\Psi_{ik}\left(\mathrm{virt}\right)\right|}^{2}-\underset{k}{\sum }{\left|\Psi_{ik}\left(\mathrm{occ}\right)\right|}^{2}$$

where Ψ*_ik_*(occ) and Ψ*_ik_*(virt) are NTO pairs for S_0_→S*_i_* transition. The electronic density’s change during T_1_→S_0_ transition was calculated in an analogous manner using canonical Kohn–Sham HSOMO-α (highest single occupied molecular α-spin orbital) and LSUMO-β (lowest single unoccupied molecular β-spin orbital).

### 3.5. Cell Culturing

The Chinese hamster ovary CHO-K1 cells were cultured in DMEM/F12 (Gibco, Carlsbad, CA, USA) medium supplemented with 10% FBS (Gibco, Carlsbad, CA, USA), 2 mM glutamine (Gibco, Carlsbad, CA, USA), and penicillin/streptomycin at a concentration of 100 U/mL (Thermo Fisher Scientific, Waltham, MA, USA), maintained in a humidified incubator at 37 °C with 5% CO_2_, and passaged routinely using trypsin-EDTA (Thermo Fisher Scientific, Waltham, MA, USA). For living-cell confocal microscopy, the cells (1 × 10^5^ CHO-K1 cells in 1.5 mL growing media) were seeded in glass-bottom 35 mm dishes (Ibidi GmbH, Gräfelfing, Germany) and incubated for 48 h until reaching a confluence of ~70%. Complexes **1**–**4** were dissolved in DMSO at a concentration of 2 mM, diluted with supplemented growing media reaching the concentration of 0.5 mM, and added to the cells in a final concentration of 5–25 µM. After incubation with the probe for 24 h, cells were washed with fresh media with all supplements.

### 3.6. MTT Assay

CHO-K1 cells were seeded in 96-well flat-bottom plates (Falcon, Corning Inc., New York, NY, USA) 1 × 10^4^ cells in 100 µL of culture medium/well and incubated overnight. The complexes were dissolved in DMSO (**1**–**3**) or water (**4**) and added to the cells at concentrations of 0–100 µM. The cells were incubated for 24 h and treated with MTT reagent 3(4,5-dimethyl-2-thiasolyl)-2,5-diphenyl-2*H*-tetrasole bromide (Thermo Fisher Scientific, Waltham, MA, USA) at the concentration of 0.5 mg/mL according to the manufacturer protocol. After further incubation at 37 °C under 5% CO_2_ for 2 h, the media was removed, and the formazan crystals were dissolved in DMSO (Merck, Munich, Germany). After incubation at 37 °C for 30 min, the absorbance was measured at 570 nm using a SPECTROstar Nano microplate reader (BMG LABTECH, Ortenberg, Germany). Viability was determined as a ratio of the average absorbance value of the wells containing conjugate to that of the control wells. The results are shown as mean ± standard deviation of 6 repetitions.

### 3.7. Lysosome Staining

LysoTracker Deep Red (LTDR, Thermo Fisher Scientific, Waltham, MA, USA) was used for the vital staining of lysosomes and late endosomes in CHO-K1 cells. After incubation with complexes **3** (5 µM 24 h) and **4** (25 µM 24 h), cells were rinsed with fresh media 3 × 1 mL and then incubated with a new portion of growing media for 15 min. LysoTracker was added to the cells for 30 min at the concentration of 50 nM prior to confocal imaging.

### 3.8. Confocal Luminescence Microscopy and PLIM Experiment

Imaging of living CHO-K1 cells was carried out by using a confocal inverted Nikon Eclipse Ti2 microscope (Nikon Corporation, Tokyo, Japan) with 60× oil immersion objective. All measurements were performed in humidified Stage Top Incubator Tokai HIT (Fujinomiya, Japan) at 37 °C and 5% CO_2_. The emission of complexes was excited with 405 nm laser. The emission was recorded in the 500–550 nm (green channel) and 570–620 nm (red channel) ranges. The fluorescence of LTDR was excited at 638 nm and recorded at 663–738 nm. Luminescent confocal microphotographs were complemented with differential interference contrast (DIC) images. The images were processed and analyzed using ImageJ software (National Institutes of Health, Bethesda, MY, USA). The quantitative co-localization analysis was performed using ImageJ JACoP Plugin to determine Pearson (P) and Manders’ (M1) co-localization coefficients. Thresholds for M1 calculation were set by a visually estimated value for each channel. Results are represented as mean ± standard deviation.

Phosphorescence lifetime imaging microscopy (PLIM) of CHO-K1 cells was carried out using a time-correlated single-photon counting (TCSPC) DCS-120 module (Becker&Hickl GmbH, Berlin, Germany) integrated into the Nikon Eclipse Ti2 confocal instrument. Emission was excited with a picosecond laser at 405 nm, phosphorescence was recorded using 575 nm long pass filter and 630/75 nm band pass filter and pinhole of 0.5–1.5. For the visualization of complex **3**, the following settings were used: frame time 7.30 s, pixel dwell time 27.30 µs, points number 1024, time per point 25.00 ns, time range of PLIM recording 25.60 µs, total acquisition time 100–140 s, and image size 512 × 512 pixels. The lifetime of **4** is higher, thus, different PLIM settings were used for it: frame time 52.75 s, pixel dwell time 200.7 µs, points number 1024, time per point 175.00 ns, time range of PLIM recording 179.20 µs, total acquisition time 130–160 s, and image size 512 × 512 pixels. Oil immersion 60× objective with zoom 5.33 provided a scan area of 0.05 mm × 0.05 mm. Phosphorescence lifetime data were processed with SPCImage 8.1 software (Becker & Hickl GmbH, Berlin, Germany) using bi- (for **4**) and tri- (for **3**) exponential decay modes with an average goodness of the fit 0.8 ≤ χ^2^ ≤ 1.2. The average number of photons per curve were not less than 5000 at binning 7–8. The colors in the PLIM images show the intensity-weighted average lifetime (τ_av_, see Equation (1)).

## 4. Conclusions

A series of neutral and ionic luminescent [Ir(**N^C**)_2_(**L^L**)] complexes based on the cyclometalating ligand containing a phenanthro-imidazole aromatic system have been synthesized and characterized. The photophysics of the **N^CH** ligand precursor and complexes in solution was studied in detail with a special accent on pH dependence of emission intensity and lifetime. DFT and TD DFT calculations were used to assign the electronic transitions responsible for the absorption and emission of these compounds in deprotonated and protonated states. Two of the obtained complexes (**3** and **4**) display clearly visible and quantitatively measurable responses of emission intensity and lifetime onto pH variations in a physiologically relevant range. These observations, together with the in vitro results of cytotoxicity assay, co-localization studies, and PLIM measurements on living cells indicate that these emitters are potentially suitable for application as pH-sensitive probes in biological systems. However, for practical applications as pH quantitative sensors, these complexes need some modification to exclude distorting effects of the typical components of the biological environment.

## Figures and Tables

**Table 1:** Photophysical properties of complexes **1** and **2**, and ligand precursor **N^CH** in CH_2_Cl_2_, complex **3** in methanol, and complex **4** in aqueous solutions, 298 K.

N°	Absorbance, nm (ε × 10^−3^, M^−1^cm^−1^)	λ_ex_, nm	λ_em_, nm	Stokes Shift *, cm^−1^	τ_av_ (aer/deg **), μs	Φ (aer/deg **), %
**N^CH** in CH_2_Cl_2_	262 (53), 280 (25), 335 (23), 363 (19)	315sh, 356sh, 372	423	3910	1.7 × 10^−3^	18
**N^CH** in CH_2_Cl_2_ + TFA (1:1)	250sh (49), 257 (63), 275 (26), 295 (19), 323 (19), 334 (19), 351 (20), 410sh (2.4)	317, 355sh (at 426 nm); 343, 377 (at 600 nm)	423, 550	750	3.1 × 10^−3^ (at 550 nm)	14
**N^CH** in CH_2_Cl_2_ + TFA conc	255 (86), 276 (31), 292sh (23), 322 (14), 334 (16), 358 (21)	335, 368	510	8355	1.1 × 10^−3^	11
**1** in CH_2_Cl_2_	261 (124), 308sh (36), 350sh (38), 364 (40), 450sh (5)	280, 308sh, 342, 364, 445	553, 596, 650sh	4140	0.53/ 4.32	2.0/25
**1** in CH_2_Cl_2_ + TFA	256, 277sh, 298sh, 320sh, 333sh, 348, 450sh	265, 325, 348, 430sh	590	5270	0.65/ 1.62	4.3/14
**1** in CH_2_Cl_2_ + Et_3_N	261 (124), 308sh (36), 350sh (38), 364 (40), 450sh (5)	280, 308sh, 342, 364, 445	554, 597, 650sh	4170	0.46/ 6.56	1.8/30
**2** in CH_2_Cl_2_	261 (149), 309sh (40), 350sh (34), 370 (41), 425sh (15)	275, 305sh, 350sh, 370, 425sh	570, 595	5990	0.69/ 6.14	2.0/17
**2** in CH_2_Cl_2_ + TFA	257, 275sh, 300sh, 310sh, 335sh, 350sh, 425sh	258, 290sh, 335sh, 350sh, 420sh	550, 585, 640sh	5350	1.02/ 6.68	3.0/21
**2** in CH_2_Cl_2_ + Et_3_N	260 (149), 309sh (40), 350sh (34), 370 (41), 425sh (15)	275, 305sh, 350sh, 370, 425sh	570, 600, 650sh	5990	0.54/ 7.37	1.9/31
**3** in CH_3_OH	258 (155), 304 (45), 322 (42), 342 (39), 357 (40), 420sh (8), 480sh (2)	256, 301sh, 321, 340, 357, 420sh, 480sh	595	4030	0.235/ 0.820	3.6/14
**3** in CH_3_OH +TFA	255 (148), 301sh (47), 325 (42), 357 (40), 420sh (9), 480sh (2)	256, 301sh, 326, 340, 357, 420sh, 480sh	650	5450	0.035/ 0.040	0.60/0.64
**3** in CH_3_OH +NEt_3_	258 (155), 304 (45), 322 (42), 342 (39), 357 (40), 420sh (8), 480sh (2)	256, 301sh, 321, 340, 357, 420sh, 480sh	595	4030	0.226/ 0.647	4.4/13
**4** in PBS pH 7.0	233 (617), 263sh (128), 271 (117), 279 (96), 308sh (24), 347sh (24), 367 (27)	285, 350sh, 367	482sh, 555, 587sh	6500	4.07/ 41.87 (at 560 nm); 1.0 × 10^−3^ (at 480 nm)	0.46/4.35
**4** in 0.1 M HCl	236sh (589), 264 (117), 271 (109), 280 (91), 337 (29), 357 (31)	274, 286, 302sh, 338, 360	563	10250	8.03/ 12.47	1.64/2.24
**4** in 0.1 M NaOH	233 (593), 263sh (122), 271 (112), 279 (91), 347sh (24), 367 (26)	285, 350sh, 367	482sh, 555, 587sh	6500	4.08/ 51.98	0.42/4.96

* Stokes shifts are calculated as the difference between the lowest energy band/shoulder in the absorption spectrum and the highest energy emission band component; ** aer—aerated solution, deg—degassed solution.

## Data Availability

Data is contained within the article and Supplementary Material.

## Supplementary Materials

The following are available online. Part 1. XRD-analysis, NMR spectroscopy and ESI mass-spectrometry data: Table S1, Figures S1–S8; Part 2. Photophysical properties of complexes 1-4 and **N^CH**: Figures S9–S15; Part 3. Computational results: Tables S2–S21, Figures S16–S25.

## Sample Availability

Samples of the compounds **1**–**4** and **N^CH** are available from the authors.
