# Supplementary material for: pH-Responsive N^C-Cyclometalated Iridium(III) Complexes: Synthesis, Photophysical Properties, Computational Results, and Bioimaging Application
Source: Molecules. 2021 Dec 30;27(1):232. doi: 10.3390/molecules27010232 (PMC8747057; doi:10.3390/molecules27010232)
Supplement: Supplementary file 1 [file molecules-27-00232-s001.zip › molecules-1540969-supplementary.pdf]

Supporting Information

for

pH-Responsive N^C-cyclometalated iridium(III) complexes: synthesis, photophysical properties, computational results, and bioimaging application

Anastasia I. Solomatina*, Daria O. Kozina, Vitaly V. Porsev*, Sergey P. Tunik*

Institute of Chemistry, St. Petersburg State University, Universitetskii av., 26, 198504 St. Petersburg, Russia; nastisol@gmail.com (A.I.S.); kozina.d@yandex.ru (D.O.K.); v.porsev@spbu.ru (V.V.P.); sergey.tunik@spbu.ru (S.P.T.)

Content

Part 1. XRD-analysis, NMR spectroscopy and ESI mass-spectrometry data 1

Part 2. Photophysical properties of complexes **1-4** and **N^CH** 10

Part 3. Computational results...14

Part 1. XRD-analysis, NMR spectroscopy and ESI mass-spectrometry data

Table S1. Selected bond distances (Å) and angles (°) in the structure of ***2****.*

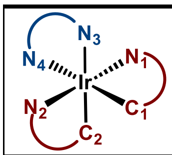					
Distances, Å		Angles, °		Angles, °	
Ir-C1	2.005(6)	N1-Ir-C1	79.7(2)	C1-Ir-N2	97.0(2)
Ir-N1	2.040(5)	N2-Ir-C2	80.2(2)	N2-Ir-N4	88.13(18)
Ir-C2	2.016(5)	N3-Ir-N4	77.1(2)	N4-Ir-N1	95.4(2)
Ir-N2	2.075(5)	C1-Ir-C2	85.7(2)	N3-Ir-N2	96.5(2)
Ir-N3	2.132(4)	C2-Ir-N1	97.2(2)	N3-Ir-N1	86.23(17)
Ir-N4	2.128(5)	C2-Ir-N4	99.3(2)	N3-Ir-C1	98.2(2)

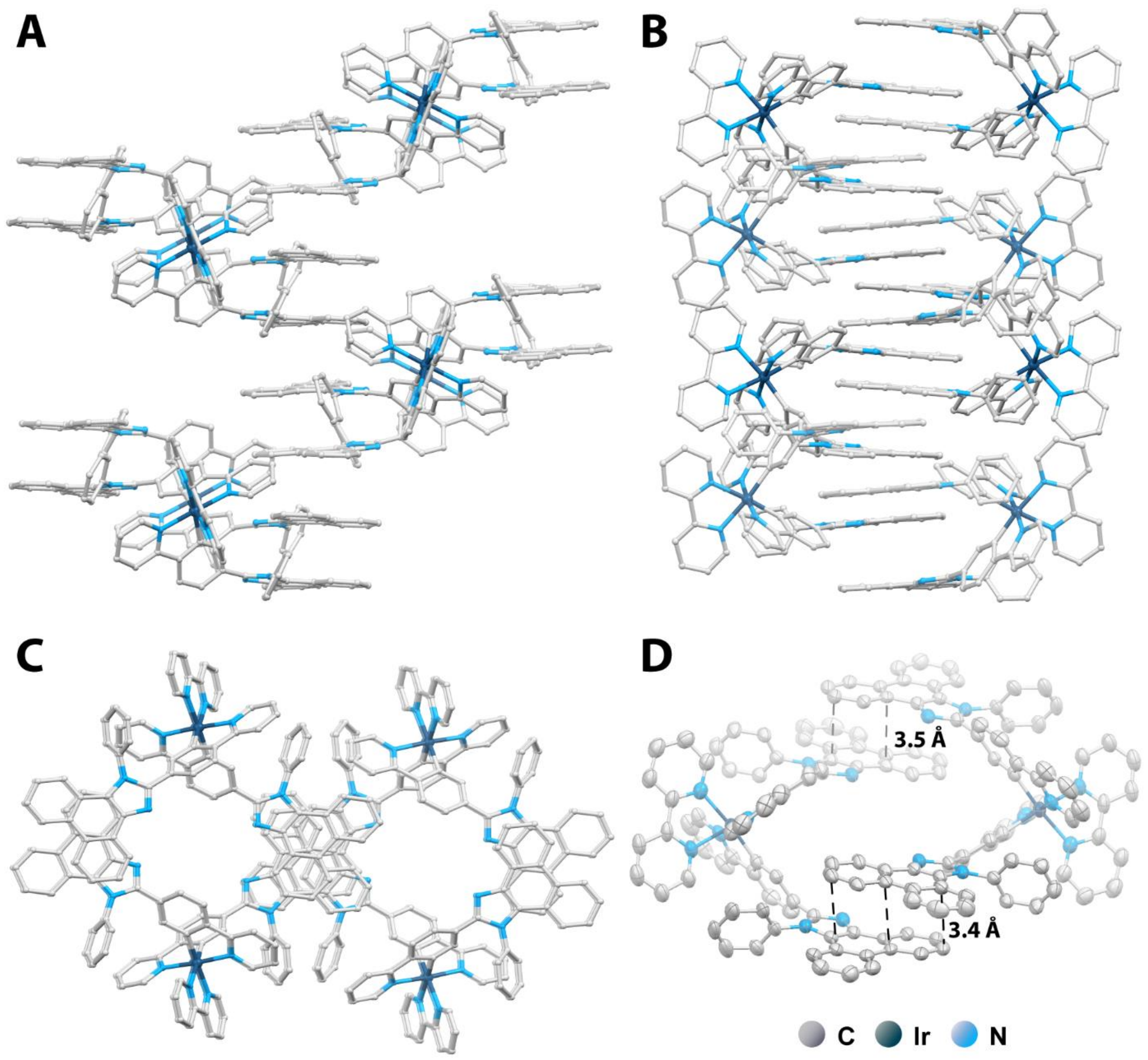

*Figure S1. Perspective view of **2** packing in the solid state: A – front view, B – right view, C – top view, D – short π-π interactions between phenanthrene moieties showing thermal ellipsoids at the 40% probability level (H-atoms are omitted for clarity).*

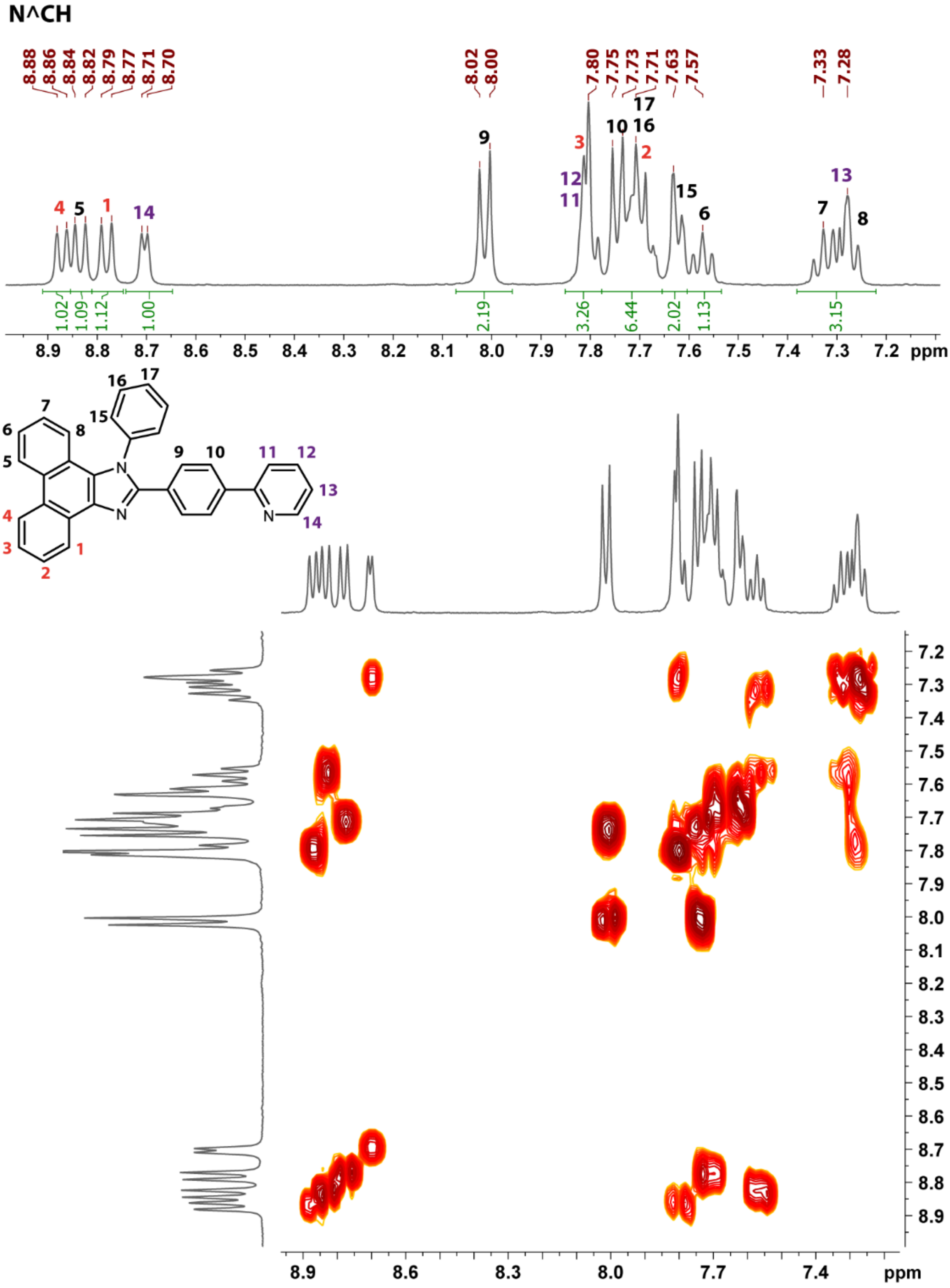

*Figure S2. ^{1}H and ^{1}H-^{1}H COSY NMR spectrum of **N^CH** in CD_2Cl_2, 298 K.*

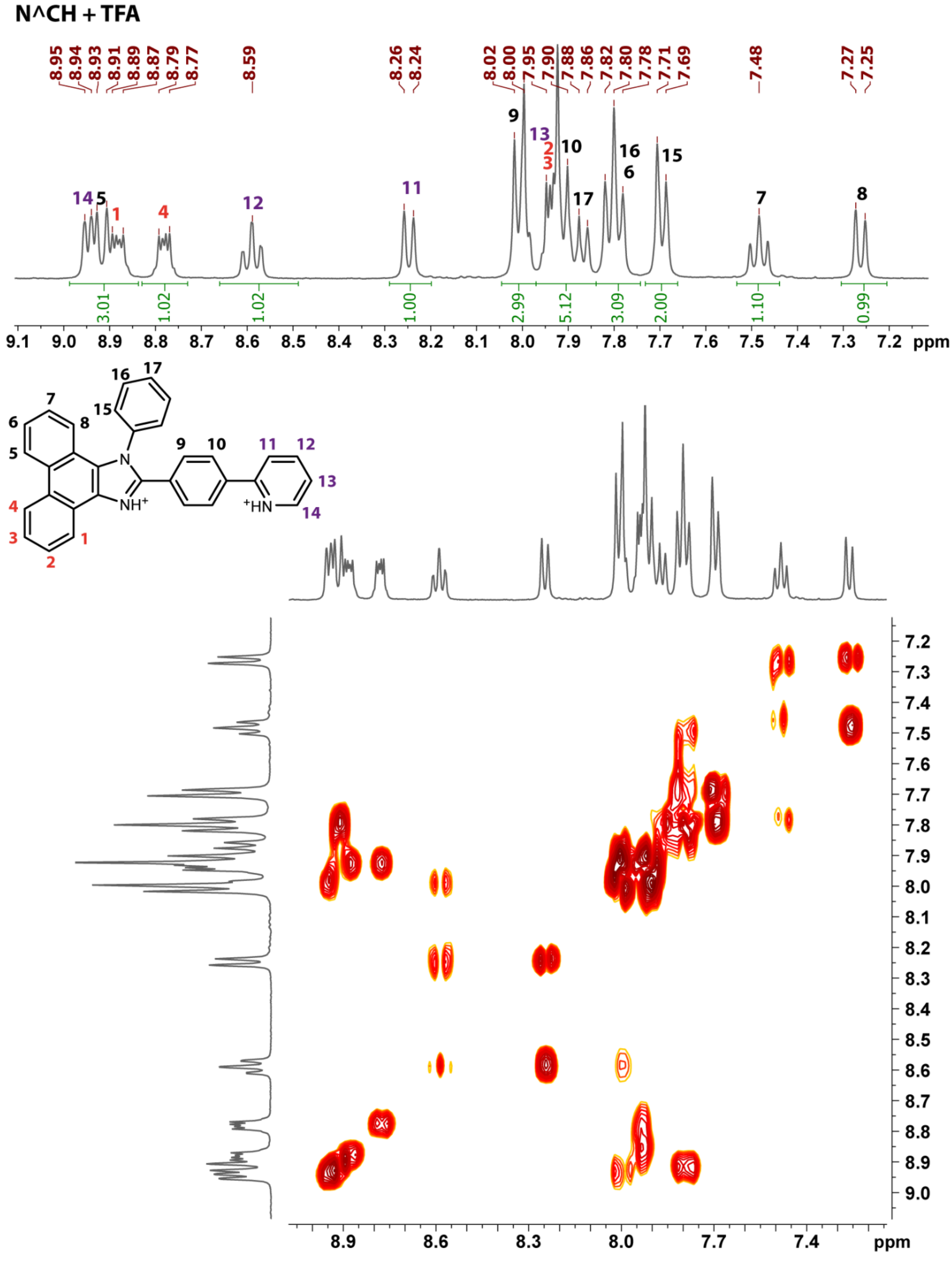

*Figure S3. ^{1}H and ^{1}H-^{1}H COSY NMR spectrum of **N^CH** in CD_2Cl_2 with TFA, 298 K.*

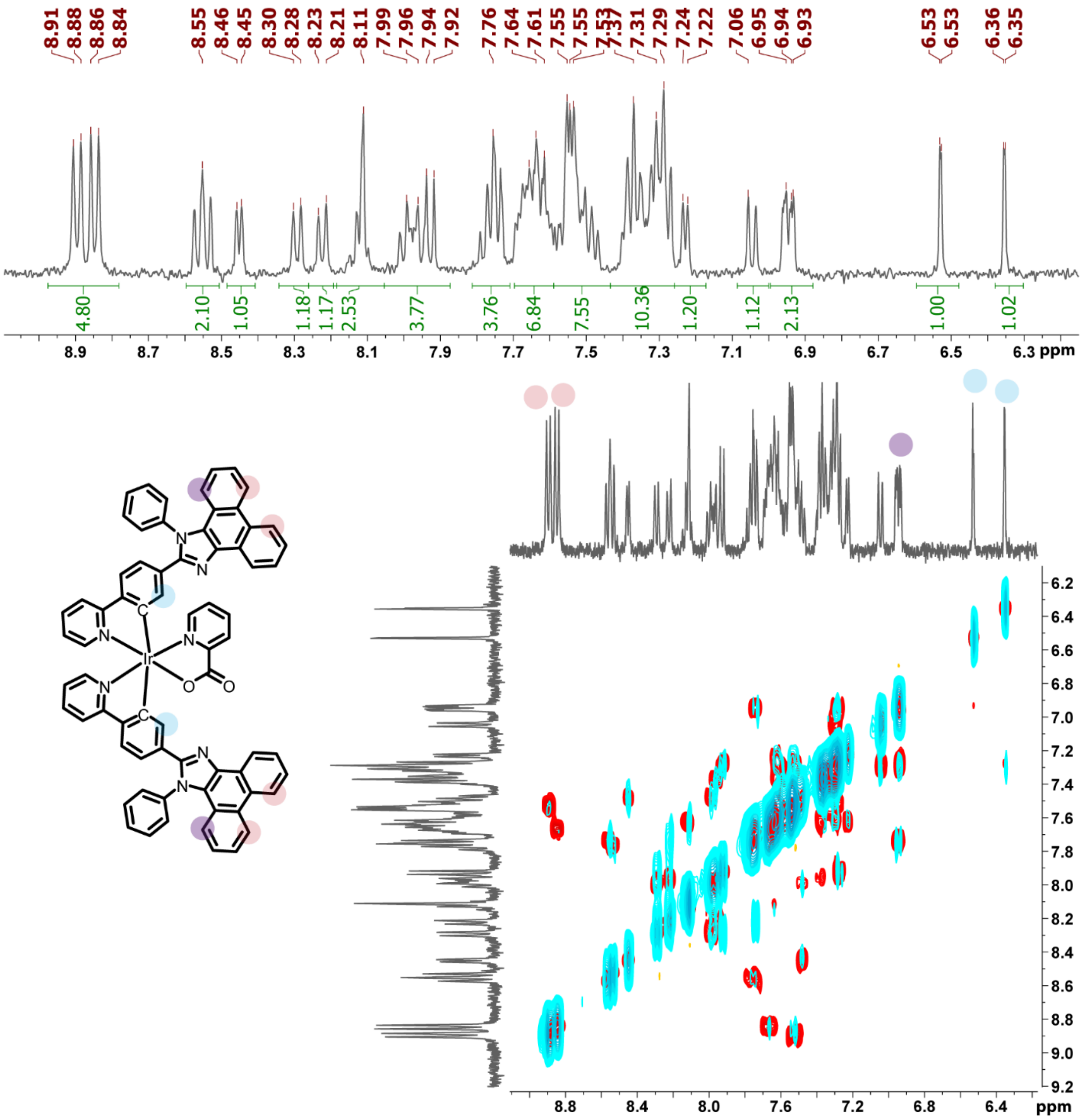

Figure S4. [1]*H and overlapped* [1]*H-*[1]*H COSY and NOESY NMR spectra of complex* ***1*** *with assignment (given by different colours at the spectrum and structural pattern) of some signals to protons, DMSO-d6, 298 K.*

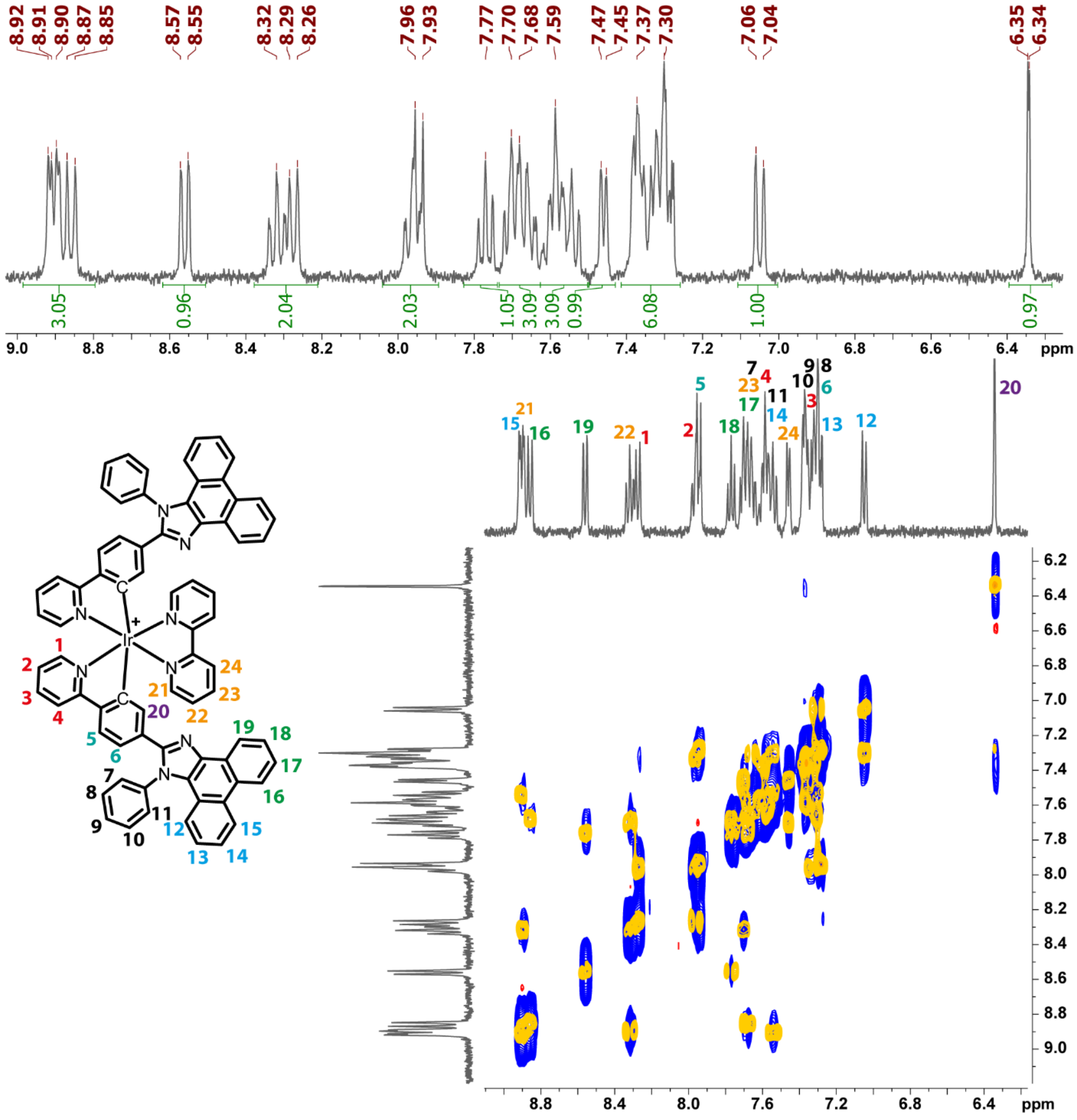

*Figure S5. ^{1}H and overlapped ^{1}H-^{1}H COSY and NOESY NMR spectra of complex **2** with full assignment of the signals to protons, DMSO-d6, 298 K.*

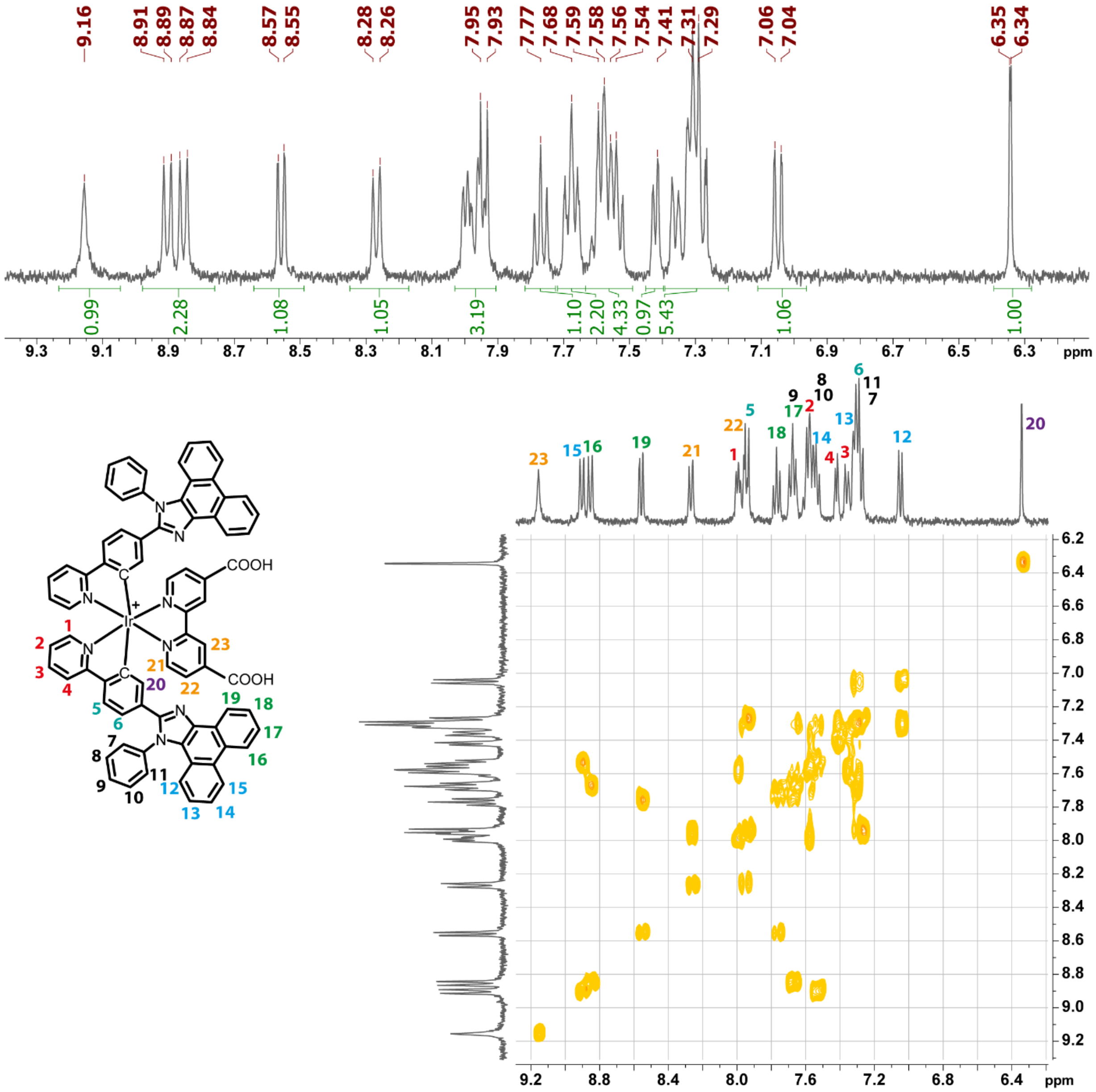

*Figure S6. ^{1}H and ^{1}H-^{1}H COSY NMR spectra of complex **3** with full assignment of the signals to protons, DMSO-d6, 298 K.*

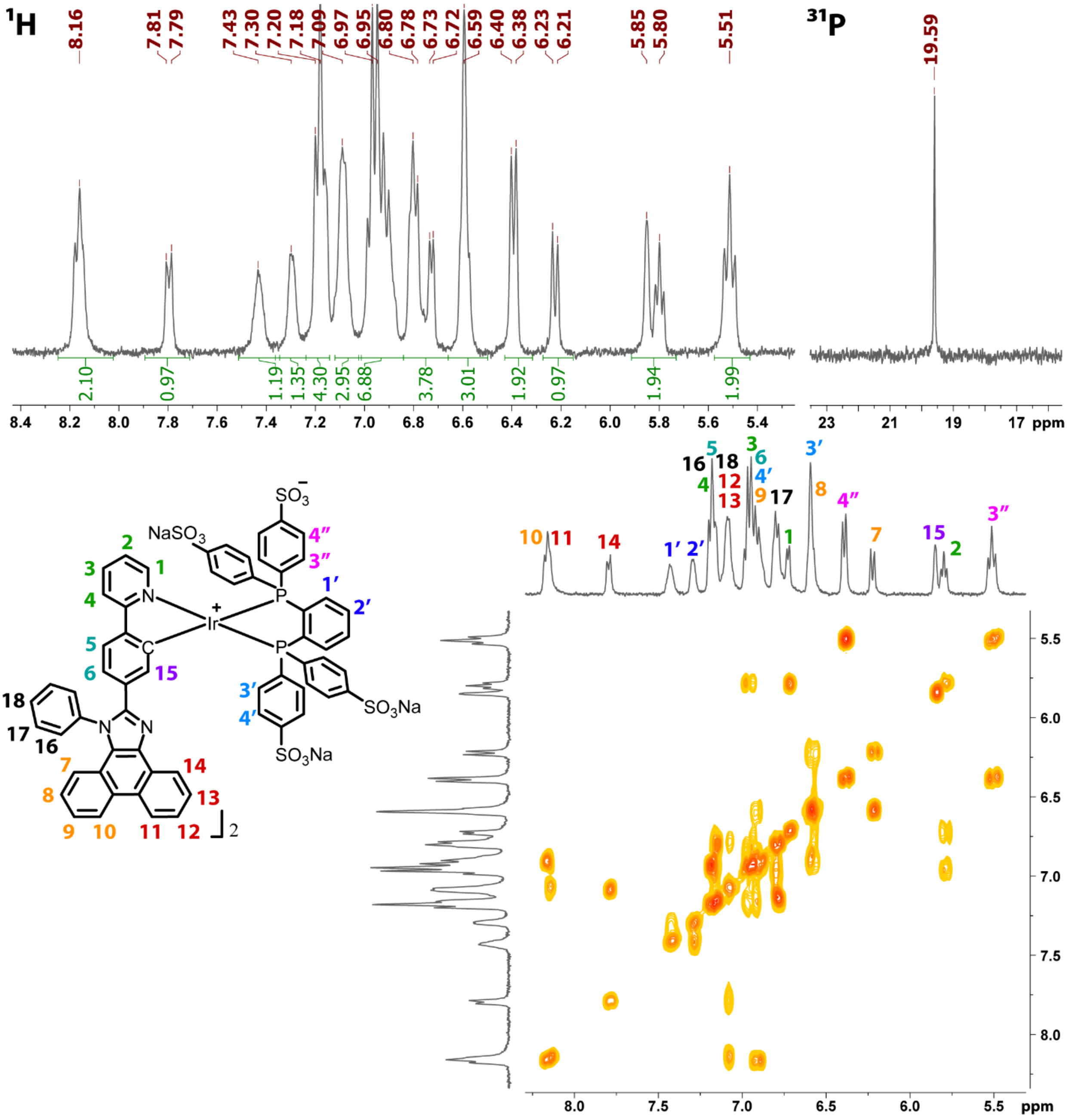

*Figure S7. ^{1}H and ^{31}P NMR spectra and ^{1}H-^{1}H COSY NMR spectra of complex **4** with full assignment of the signals to protons, DMSO-d6, 298 K.*

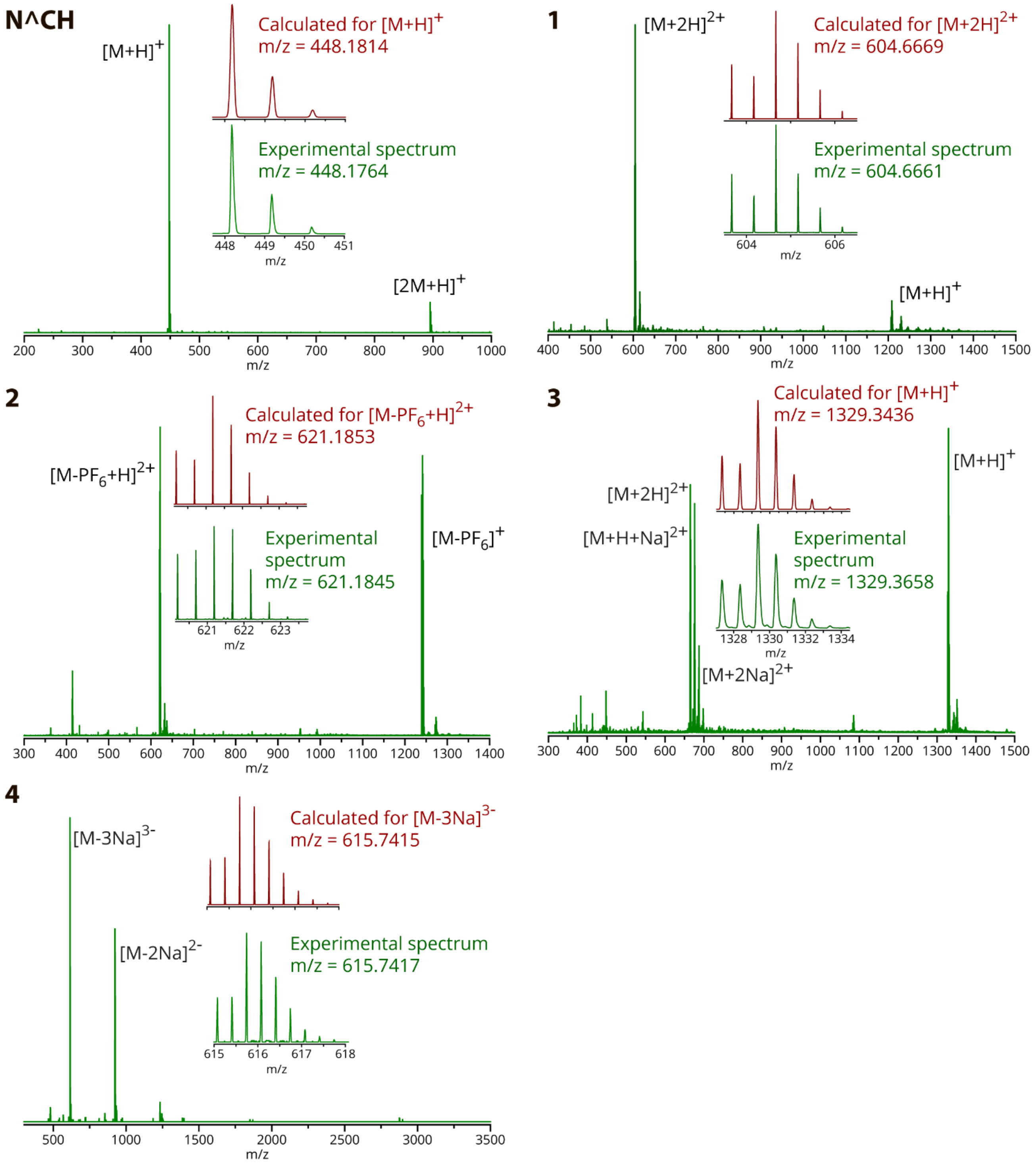

*Figure S8. Experimental and calculated ESI mass spectra of **1-4** and **N^CH**.*

Part 2. Photophysical properties of complexes 1-4 and N^CH

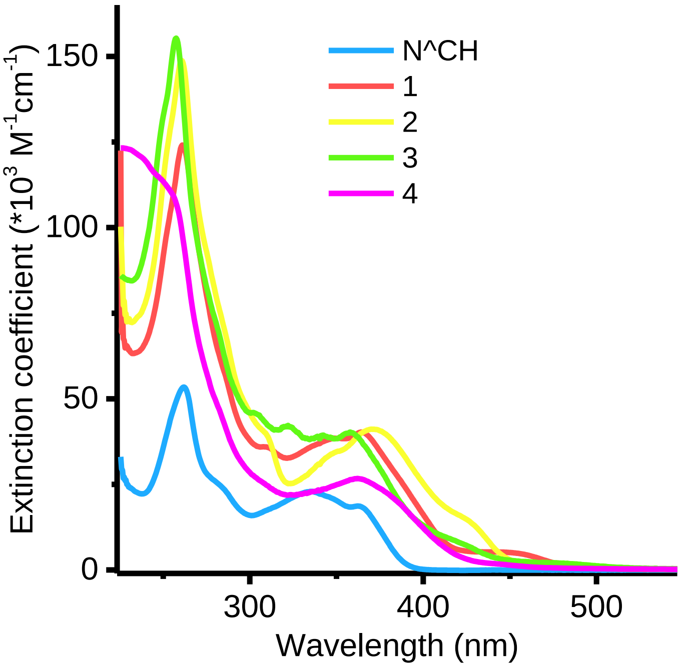

Figure S9. Absorption spectra of ***N^CH*** *and complexes* ***1, 2*** *in CH_2Cl_2,* ***3*** *in CH_3OH, and* ***4*** *in water, 298 K.*

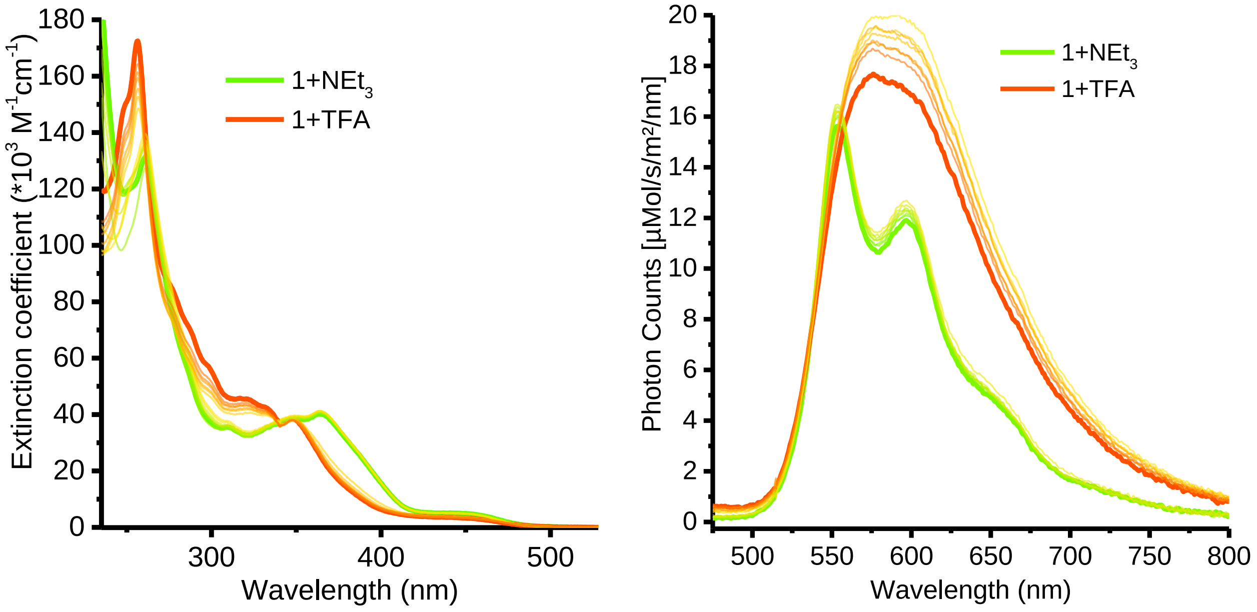

Figure S10. Absorption (left) and emission (right) spectra of complex ***1*** *in CH_2Cl_2 upon addition of base (NEt_3, green) or acid (THF, red), 298 K.*

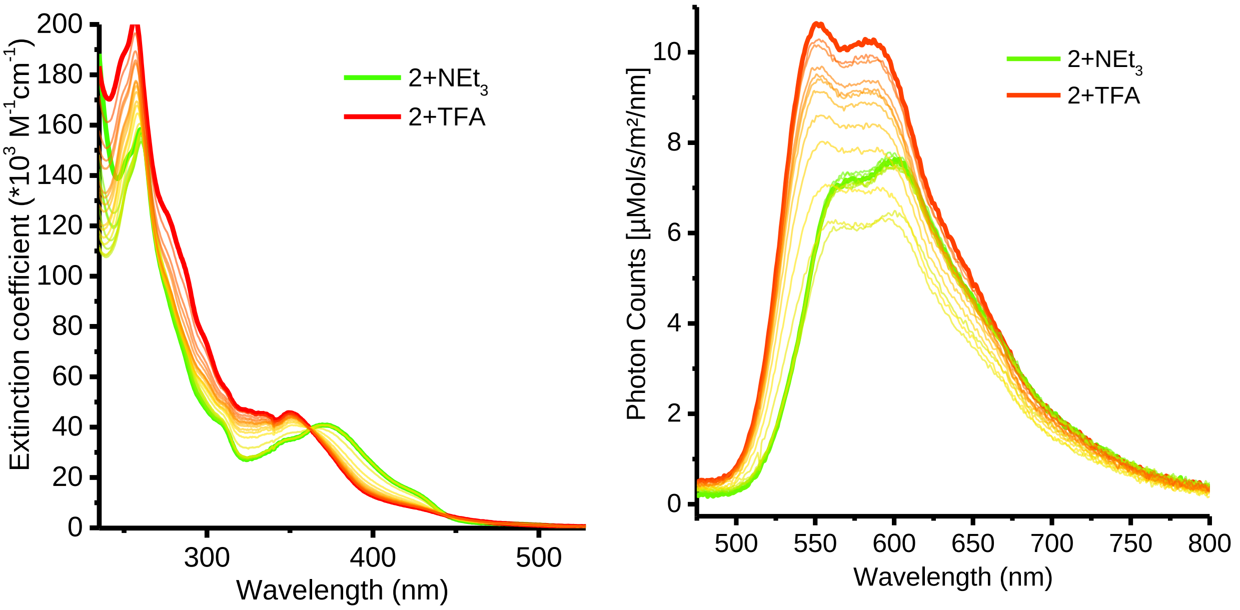

*Figure S11. Absorption (left) and emission (right) spectra of complex **2** in CH_2Cl_2 upon addition of base (NEt_3, green) or acid (THF, red), 298 K.*

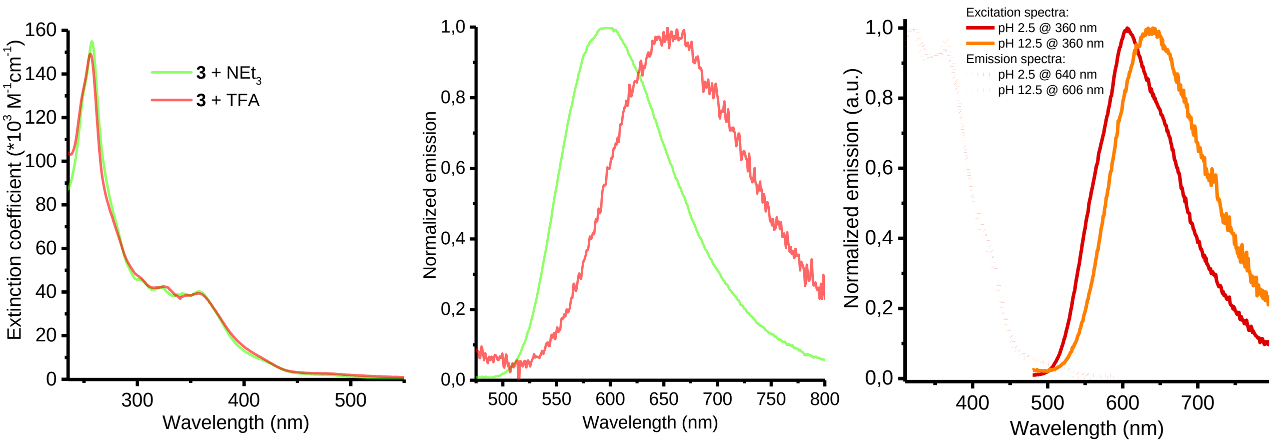

*Figure S12. Absorption (left) and emission (center) spectra of complex **3** in MeOH upon addition of base (NEt_3, green) or acid (TFA, red). Right: excitation (dashed) and emission (solid) spectra of complex 3 in aqueous buffer solutions at pH 2.5 and 12.5, 298 K, aerated solutions.*

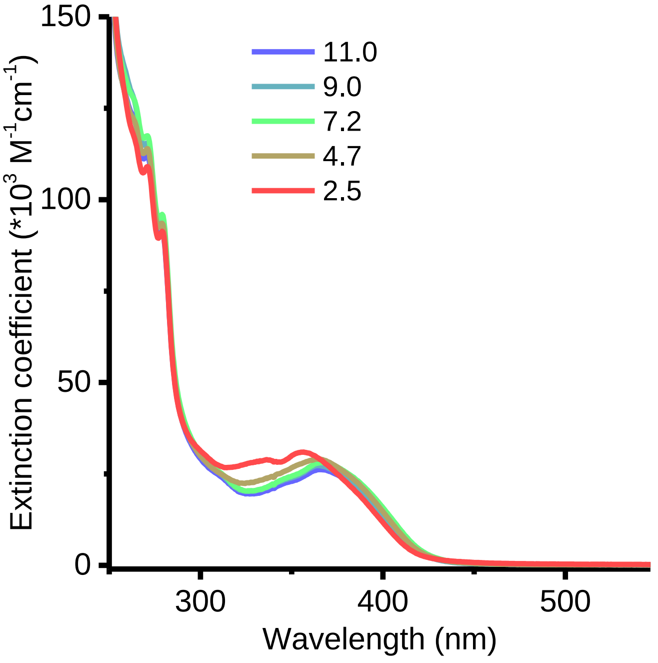

*Figure S13. Absorption spectra of **4** in aqueous buffer solutions with different pH, 298 K.*

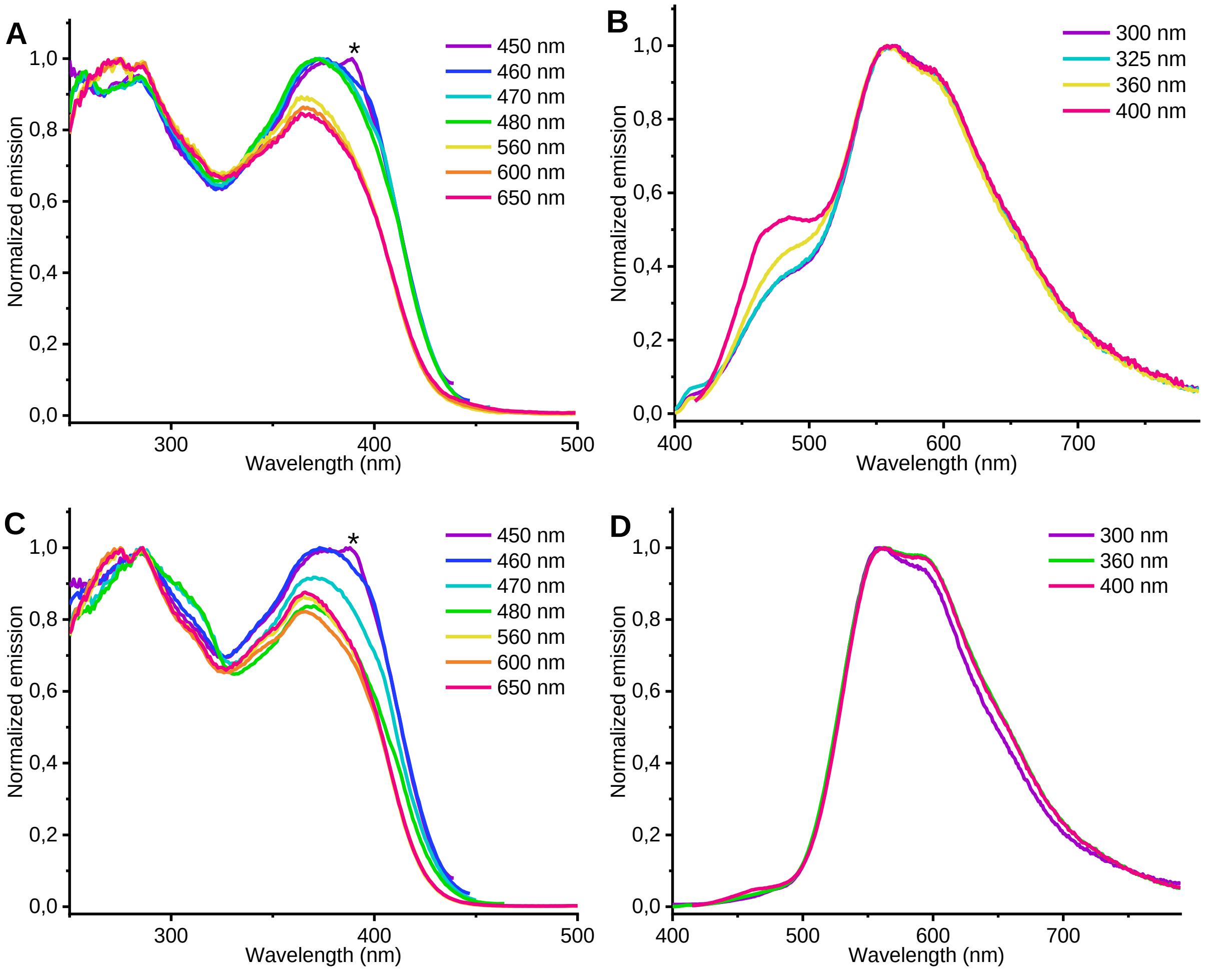

*Figure S14. Excitation (A, C) and emission (B, D) spectra of **4** in 0.1 M NaOH aqueous air-saturated (A, B) and Ar-saturated (C, D) solutions, 298 K. The asterisks denote the Raman peak of water.*

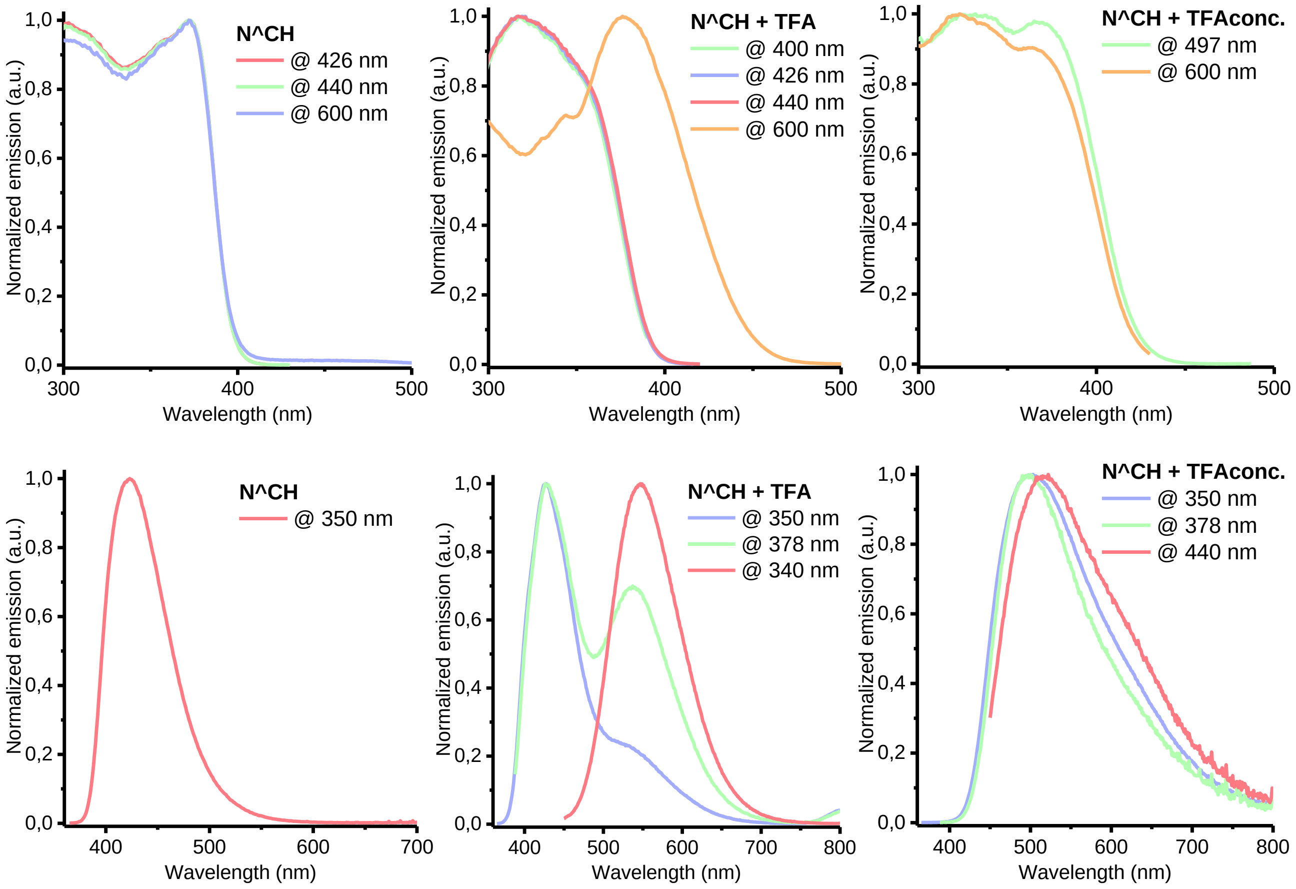

Figure S15. Excitation (top) and emission (bottom) spectra of ***N^CH*** *in CH_2Cl_2 upon addition of TFA, 298 K.*

Part 3. Computational results

*Table S2. Experimental and calculated photophysical properties of **N^CH** and complexes **1** and **2** in CH_2Cl_2, complex **3** in CH_3OH, and complex **4** in water.*

Complex	Abs, nm (ε*10^{-4}, L*mol^{-1}*cm^{-1})		λ_{em}, nm	
	exp	calc	exp	calc
**1**	261 (124), 308sh (36), 350sh (38), 364 (40), 450sh (5)	255 (139), 288sh (65), 373 (55), 427sh (19)	554, 597, 650sh	534
{**1**+2H$^+$}	256, 277sh, 298sh, 320sh, 333sh, 348, 450sh	250 (121), 279sh (68), 357 (75), 437sh (15)	590	559
**2**	260 (149), 309sh (40), 350sh (34), 370 (41), 425sh (15)	255 (172), 288sh (75), 320sh (35), 395 (50)	570, 600, 650sh	517
{**2**+2H$^+$}	257, 275sh, 300sh, 310sh, 335sh, 350sh, 425sh	249 (151), 275sh (86), 293sh (67), 364 (73), 408sh (25)	550, 585, 640sh	484
**4**	264sh (122), 270 (112), 279 (91), 308sh (25), 366 (26)	254 (186), 296sh (46), 317sh (31), 393 (72)	482sh, 555, 587sh	531
{**4**+2H$^+$}	264sh (117), 271 (110), 279 (91), 356 (31)	250 (148), 284 (60), 373 (69)	563	536
{**3**−2H$^+$}	258 (155), 304 (45), 322 (42), 342 (39), 357 (40), 420sh (8), 480sh (2)	253 (194), 288sh (72), 335sh (30), 357sh (46), 373 (61), 401sh (36)	595	466
{**3**+2H$^+$}	255 (148), 301sh (47), 325 (42), 357 (40), 420sh (9), 480sh (2)	250 (126), 287 (74), 359 (71)	650	556
**N^CH**	262 (53), 280 (25), 335 (23), 363 (19)	254 (59), 268 (55), 291sh (25), 352 (56)	423	406

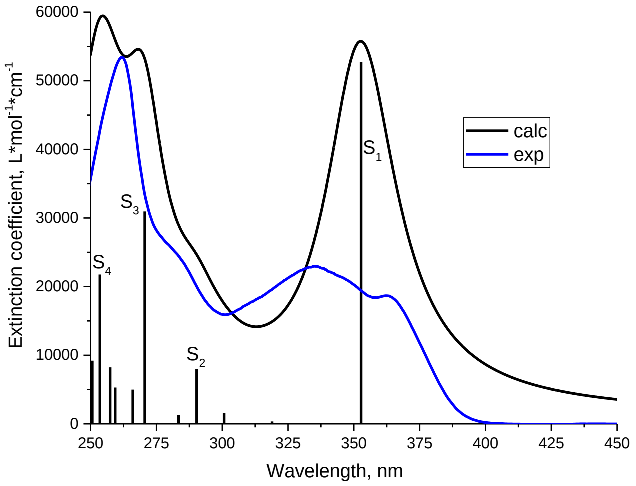

*Figure S16. Absorption spectra of **N^CH** in CH_2Cl_2 solution: experimental (blue) and calculated (black) lines with oscillator strengths of electronic transitions (bars).*

*Table S3. Experimental and calculated absorption maxima (λ), extinction coefficients (ε), oscillator strengths (f) of **N^CH.***

λ_{abs}, nm (exp)	ε*10^{-3}, L*mol^{-1}*cm^{-1} (exp)	Transitions	λ_{abs}, nm (calc)	f (calc)	Contribution of main NTO pair in transition (%)
261	53	S_0-S_4	254	0.38	43
		S_0-S_3	271	0.55	85
284sh	24	S_0-S_2	290	0.14	52
335	23	S_0-S_1	353	0.93	97

*Table S4 The decrease (blue) and increase (red) in electron density for most intensive electronic absorption transitions of **N^CH**. The data for the corresponding interfragment charge transfer (IFCT) are given below the figures. Diagonal values represent intraligand transitions, off-diagonal values represent a charge transfer from "Donor" to "Acceptor". Fragments: PhPy – 2-phenylpyridine, Im – imidazole ring, phen – phenanthrene, Ph – phenyl ring.*

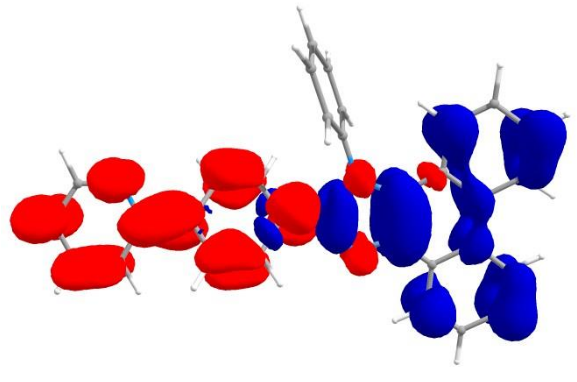

$S_0 \rightarrow S_1$

Donor	Acceptor			
	PhPy	Im	Ph	phen
PhPy	0.119	0.024	0.002	0.016
Im	0.310	0.062	0.006	0.041
Ph	0.004	0.001	0.000	0.001
phen	0.307	0.062	0.005	0.041

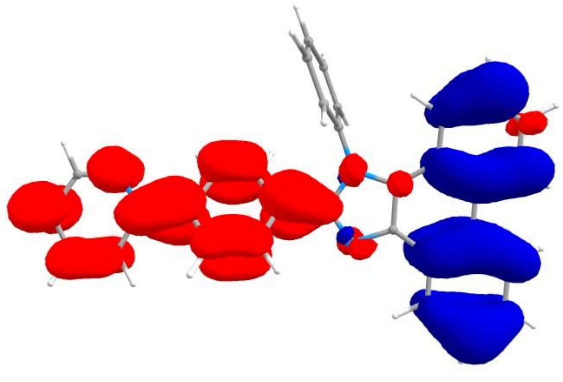

$S_0 \rightarrow S_2$

Donor	Acceptor			
	PhPy	Im	Ph	phen
PhPy	0.036	0.009	0.022	0.009
Im	0.107	0.027	0.067	0.026
Ph	0.003	0.001	0.002	0.001
phen	0.325	0.082	0.204	0.080

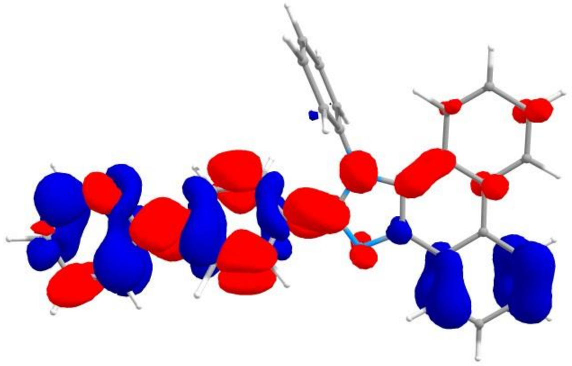

$S_0 \rightarrow S_3$

Donor	Acceptor			
	PhPy	Im	Ph	phen
PhPy	0.503	0.105	0.011	0.080
Im	0.047	0.010	0.001	0.007
Ph	0.013	0.003	0.000	0.002
phen	0.156	0.033	0.003	0.025

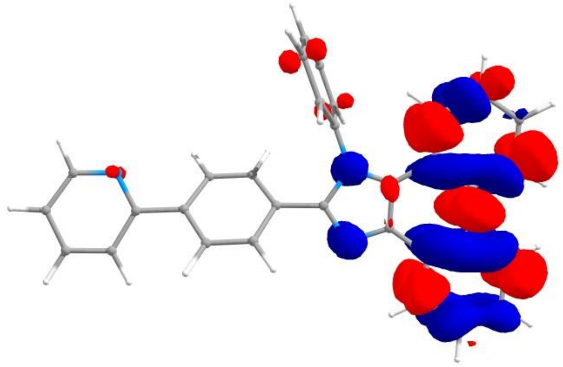

$S_0 \rightarrow S_4$

Donor	Acceptor			
	PhPy	Im	Ph	phen
PhPy	0.011	0.003	0.002	0.019
Im	0.047	0.013	0.008	0.081
Ph	0.054	0.015	0.009	0.093
phen	0.204	0.057	0.034	0.351

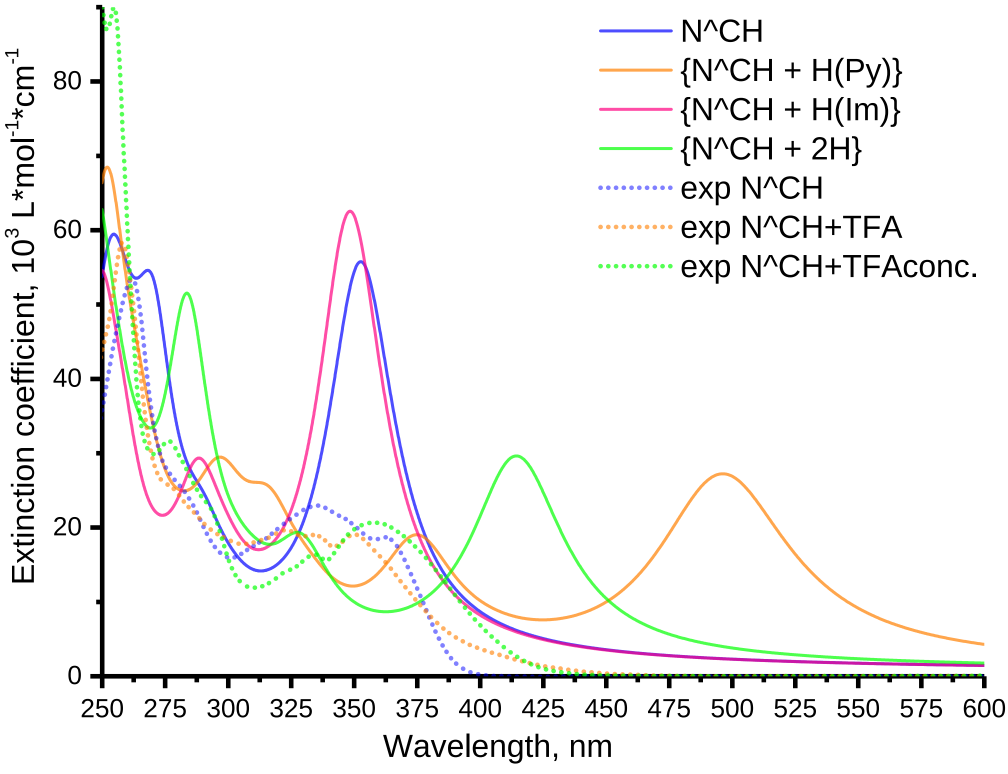

*Figure S17. Experimental (dashed lines) and calculated (solid lines) absorption spectra of **N^CH** and products of its protonation on pyridine {**N^CH** + H^+(Py)}, imidazole {**N^CH** + H^+(Im)}, and on both moieties {**N^CH** + $2H^+$} in CH_2Cl_2.*

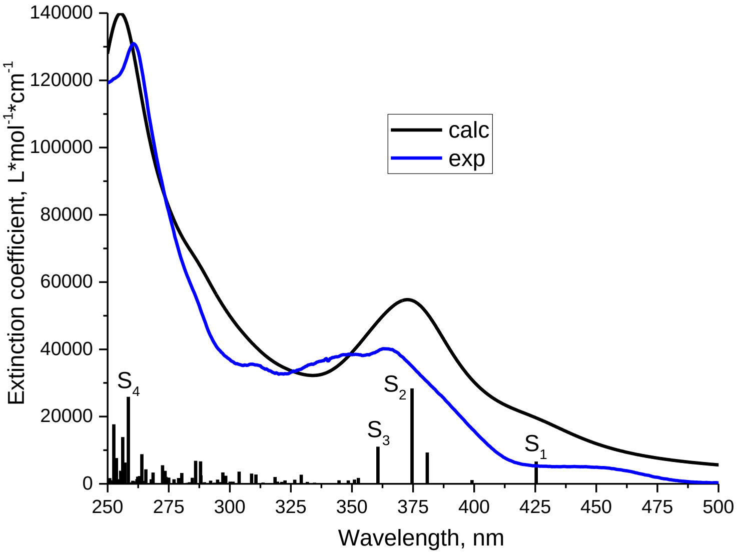

*Figure S18. Absorption spectra of complex **1** in CH_2Cl_2 solution: experimental (blue) and calculated (black) lines with oscillator strengths of electronic transitions (bars).*

*Table S5. Experimental and calculated absorption maxima (λ), extinction coefficients (ε), oscillator strengths (f) of **1**.*

Complex	λ_{abs}, nm (exp)	ε*10^{-3}, L*mol^{-1}*cm^{-1} (exp)	Transitions	λ_{abs}, nm (calc)	*f* (calc)	Contribution of main NTO pair in transition (%)
**1**	261	124	S_0-S_4	258	0.53	39
	364	40	S_0-S_3	361	0.22	87
			S_0-S_2	375	0.58	90
	450sh	5	S_0-S_1	425	0.14	95

*Table S6. The decrease (blue) and increase (red) in electron density for most intensive electronic absorption transitions of **1**. The data for the corresponding interfragment charge transfer (IFCT) are given below the figures. Diagonal values represent intraligand transitions, off-diagonal values represent a charge transfer from "Donor" to "Acceptor".*

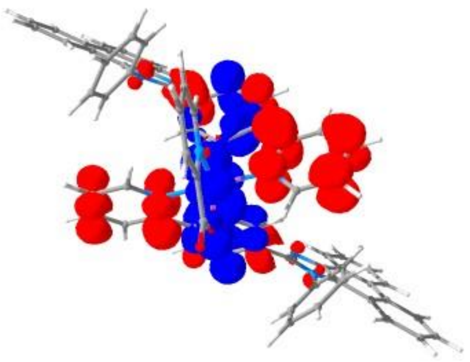

$S_0 \rightarrow S_1$

Donor	Acceptor			
	Ir	Pic	N-C	N-C
Ir	0.016	0.004	0.153	0.223
Pic	0.002	0.000	0.016	0.024
N-C	0.012	0.003	0.114	0.167
N-C	0.011	0.002	0.102	0.150

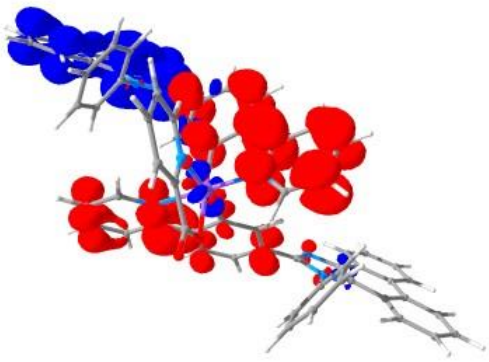

$S_0 \rightarrow S_2$

Donor	Acceptor			
	Ir	Pic	N-C	N-C
Ir	0.001	0.000	0.009	0.019
Pic	0.000	0.000	0.001	0.002
N-C	0.004	0.001	0.033	0.067
N-C	0.036	0.010	0.267	0.549

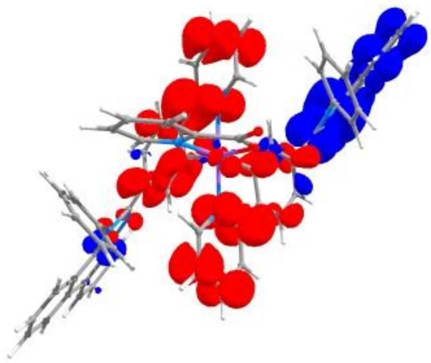

$S_0 \rightarrow S_3$

Donor	Acceptor			
	Ir	Pic	N-C	N-C
Ir	0.001	0.000	0.008	0.008
Pic	0.000	0.000	0.001	0.001
N-C	0.029	0.016	0.346	0.374
N-C	0.008	0.005	0.098	0.106

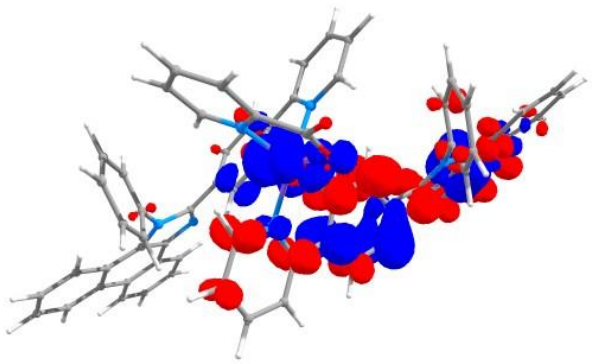

$S_0 \rightarrow S_4$

Donor	Acceptor			
	Ir	Pic	N-C	N-C
Ir	0.006	0.006	0.110	0.039
Pic	0.001	0.001	0.012	0.004
N-C	0.020	0.020	0.401	0.141
N-C	0.008	0.008	0.166	0.058

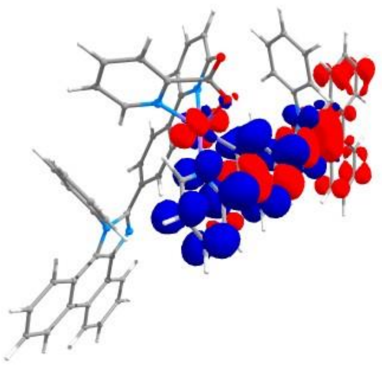

$T_1 \rightarrow S_0$

Donor	Acceptor			
	Ir	Pic	N-C	N-C
Ir	0.003	0.000	0.034	0.001
Pic	0.001	0.000	0.007	0.000
N-C	0.066	0.005	0.848	0.022
N-C	0.001	0.000	0.013	0.000

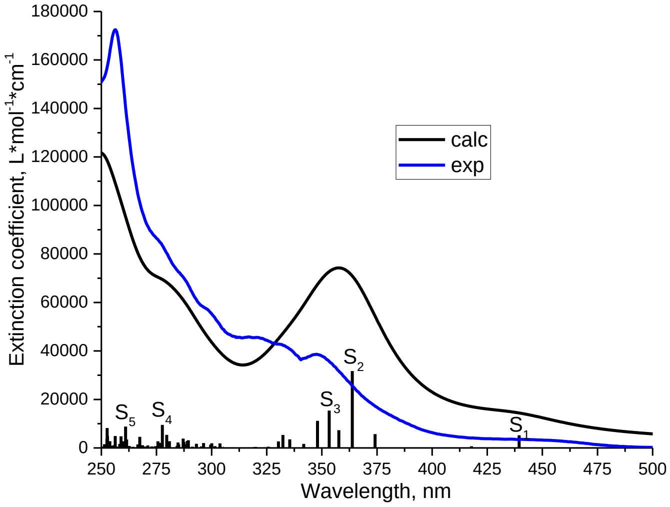

*Figure S19. Absorption spectra of {**1**+2H$^+$} in CH_2Cl_2 solution: experimental (blue) and calculated (black) lines with oscillator strengths of electronic transitions (bars).*

*Table S7. Table 1. Experimental and calculated absorption maxima (λ), extinction coefficients (ε), oscillator strengths (f) {**1**+2H$^+$}.*

Complex	λ_{abs}, nm (exp)	ε*10^{-3}, L*mol^{-1}*cm^{-1} (exp)	Transitions	λ_{abs}, nm (calc)	*f* (calc)	Contribution of main NTO pair in transition (%)
{**1**+2H$^+$}	256	172	S_0-S_5	261	0.18	28
	277sh	84	S_0-S_4	278	0.19	59
	348	39	S_0-S_3	353	0.31	63
			S_0-S_2	364	0.65	61
	450sh	4	S_0-S_1	439	0.11	95

*Table S8. The decrease (blue) and increase (red) in electron density for most intensive electronic absorption transitions of {**1**+2H$^+$}. The data for the corresponding interfragment charge transfer (IFCT) are given below the figures. Diagonal values represent intraligand transitions, off-diagonal values represent a charge transfer from "Donor" to "Acceptor".*

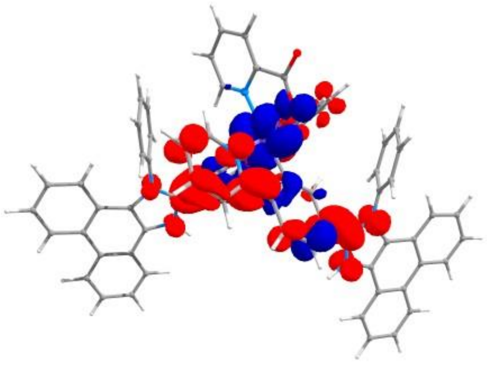

$S_0 \rightarrow S_1$

Donor	Acceptor			
	Ir	Pic	N-C	N-C
Ir	0.013	0.001	0.247	0.157
Pic	0.002	0.000	0.030	0.019
N-C	0.009	0.001	0.164	0.104
N-C	0.008	0.001	0.151	0.096

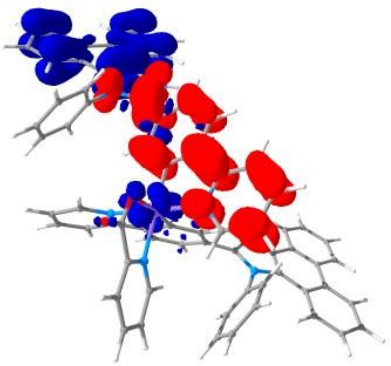

$S_0 \rightarrow S_2$

Donor	Acceptor			
	Ir	Pic	N-C	N-C
Ir	0.007	0.001	0.157	0.082
Pic	0.002	0.000	0.043	0.023
N-C	0.017	0.002	0.401	0.210
N-C	0.001	0.000	0.034	0.018

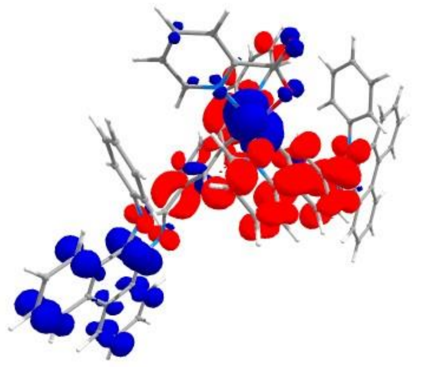

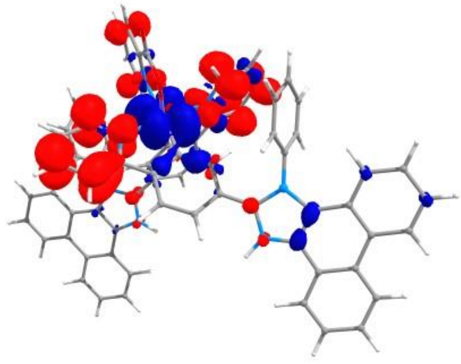

$S_0 \rightarrow S_3$

Donor	Acceptor			
	Ir	Pic	N-C	N-C
Ir	0.012	0.001	0.214	0.184
Pic	0.003	0.000	0.049	0.042
N-C	0.004	0.000	0.065	0.056
N-C	0.011	0.001	0.193	0.166

$S_0 \rightarrow S_4$

Donor	Acceptor			
	Ir	Pic	N-C	N-C
Ir	0.010	0.055	0.210	0.214
Pic	0.002	0.010	0.040	0.041
N-C	0.004	0.023	0.088	0.089
N-C	0.004	0.024	0.092	0.094

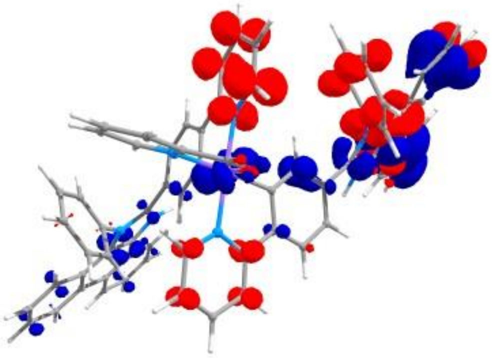

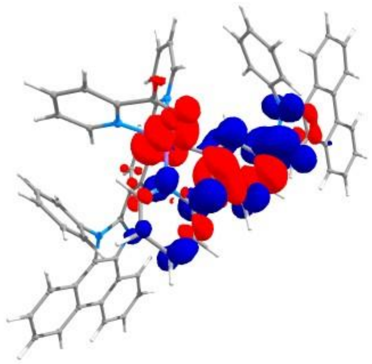

$S_0 \rightarrow S_5$

Donor	Acceptor			
	Ir	Pic	N-C	N-C
Ir	0.002	0.002	0.073	0.020
Pic	0.000	0.000	0.014	0.004
N-C	0.012	0.012	0.511	0.141
N-C	0.004	0.004	0.158	0.044

$T_1 \rightarrow S_0$

Donor	Acceptor			
	Ir	Pic	N-C	N-C
Ir	0.007	0.001	0.020	0.002
Pic	0.000	0.000	0.001	0.000
N-C	0.218	0.032	0.635	0.049
N-C	0.008	0.001	0.024	0.002

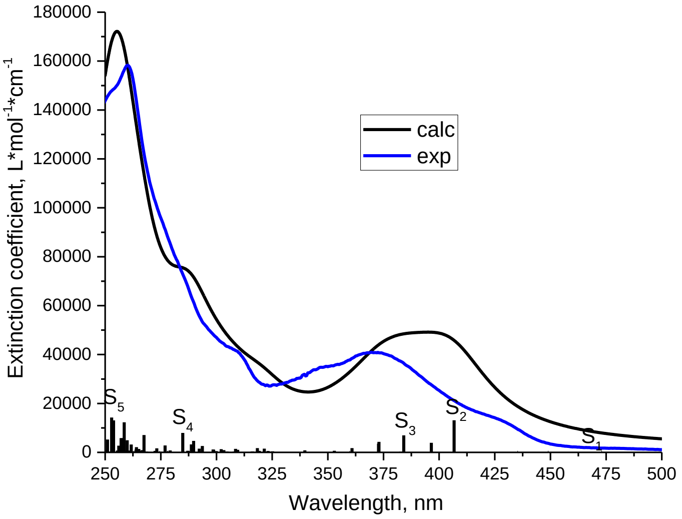

*Figure S20. Absorption spectra of complex **2** in CH_2Cl_2 solution: experimental (blue) and calculated (black) lines with oscillator strengths of electronic transitions (bars).*

*Table S9. Experimental and calculated absorption maxima (λ), extinction coefficients (ε), oscillator strengths (f) of **2.***

Complex	λ_{abs}, nm (exp)	$\varepsilon*10^{-3}$, $L*mol^{-1}*cm^{-1}$ (exp)	Transitions	λ_{abs}, nm (calc)	*f* (calc)	Contribution of main NTO pair in transition (%)
**2**	260	149	S_0-S_5	253	0.48	35
	309sh	40	S_0-S_4	285	0.27	45
	370	41	S_0-S_3	384	0.24	92
	425sh	15	S_0-S_2	407	0.44	97
			S_0-S_1	473	0	99

*Table S10. The decrease (blue) and increase (red) in electron density for most intensive electronic absorption transitions of **2**. The data for the corresponding interfragment charge transfer (IFCT) are given below the figures. Diagonal values represent intraligand transitions, off-diagonal values represent a charge transfer from "Donor" to "Acceptor".*

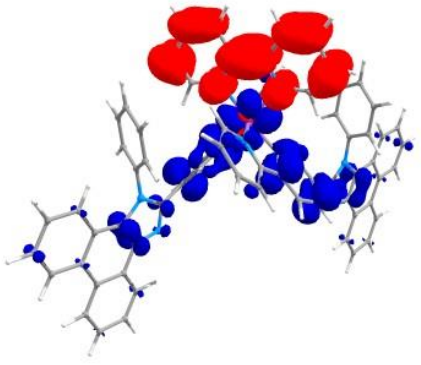

$S_0 \rightarrow S_1$

Donor	Acceptor			
	Ir	NN	N-C	N-C
Ir	0.009	0.244	0.001	0.001
NN	0.001	0.015	0.000	0.000
N-C	0.013	0.348	0.002	0.002
N-C	0.013	0.349	0.002	0.002

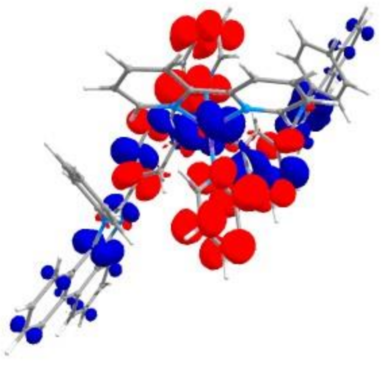

$S_0 \rightarrow S_2$

Donor	Acceptor			
	Ir	NN	N-C	N-C
Ir	0.008	0.001	0.086	0.086
NN	0.001	0.000	0.006	0.006
N-C	0.018	0.002	0.191	0.191
N-C	0.018	0.002	0.192	0.192

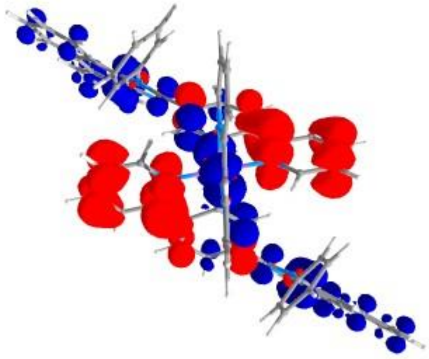

$S_0 \rightarrow S_3$

Donor	Acceptor			
	Ir	NN	N-C	N-C
Ir	0.009	0.001	0.095	0.095
NN	0.001	0.000	0.006	0.006
N-C	0.018	0.002	0.187	0.187
N-C	0.018	0.002	0.187	0.187

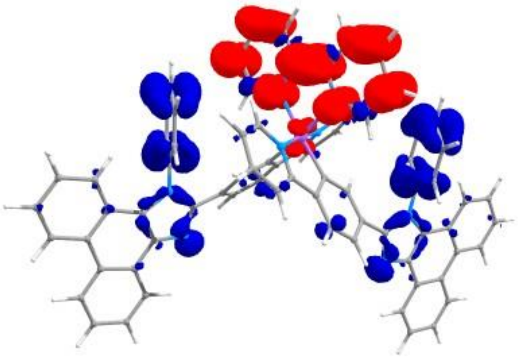

$S_0 \rightarrow S_4$

Donor	Acceptor			
	Ir	NN	N-C	N-C
Ir	0.008	0.203	0.025	0.025
NN	0.004	0.099	0.012	0.012
N-C	0.009	0.237	0.030	0.030
N-C	0.009	0.237	0.030	0.030

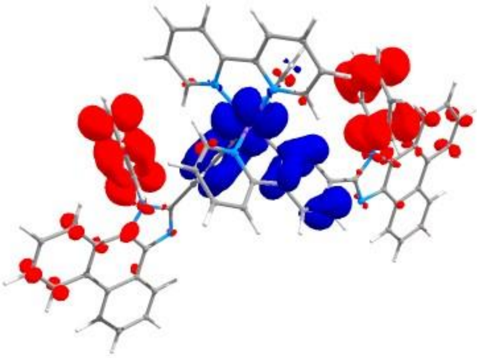

$S_0 \rightarrow S_5$

Donor	Acceptor			
	Ir	NN	N-C	N-C
Ir	0.002	0.007	0.076	0.076
NN	0.000	0.000	0.005	0.005
N-C	0.005	0.018	0.196	0.195
N-C	0.005	0.018	0.196	0.195

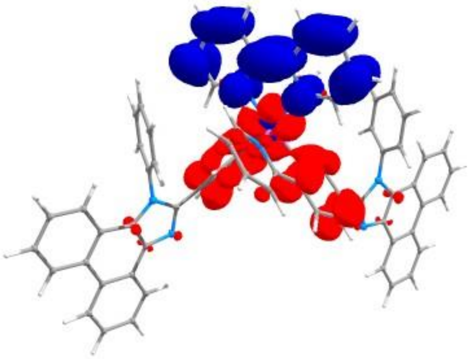

$T_1 \rightarrow S_0$

Donor	Acceptor			
	Ir	NN	N-C	N-C
Ir	0.011	0.001	0.012	0.012
NN	0.291	0.023	0.320	0.320
N-C	0.001	0.000	0.002	0.002
N-C	0.001	0.000	0.002	0.002

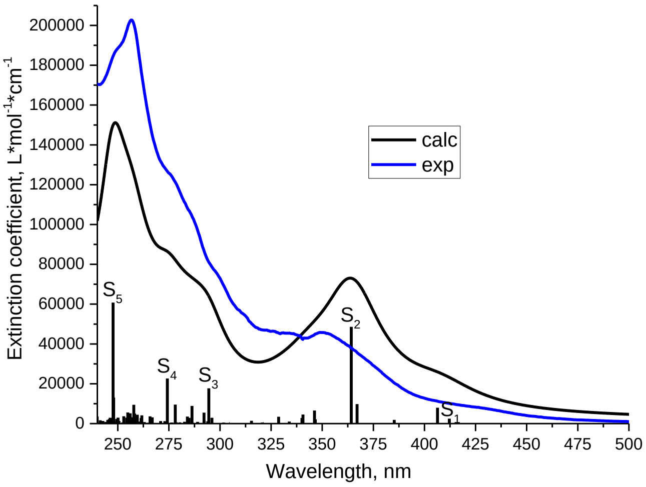

*Figure S21. Absorption spectra of {**2**+2H$^+$} in CH_2Cl_2 solution: experimental (blue) and calculated (black) lines with oscillator strengths of electronic transitions (bars).*

*Table S11. Experimental and calculated absorption maxima (λ), extinction coefficients (ε), oscillator strengths (f) {**2**+2H$^+$}.*

Complex	λ_{abs}, nm (exp)	ε*10^{-3}, L*mol^{-1}*cm^{-1} (exp)	Transitions	λ_{abs}, nm (calc)	*f* (calc)	Contribution of main NTO pair in transition (%)
{**2**+2H$^+$}	257	202	S_0-S_5	248	1.07	33
	275sh	124	S_0-S_4	274	0.40	36
	300sh	72	S_0-S_3	295	0.31	74
	350	46	S_0-S_2	364	0.86	75
	425sh	8	S_0-S_1	412	0.04	96

*Table S12. The decrease (blue) and increase (red) in electron density for most intensive electronic absorption transitions of {**2**+2H$^+$}. The data for the corresponding interfragment charge transfer (IFCT) are given below the figures. Diagonal values represent intraligand transitions, off-diagonal values represent a charge transfer from "Donor" to "Acceptor".*

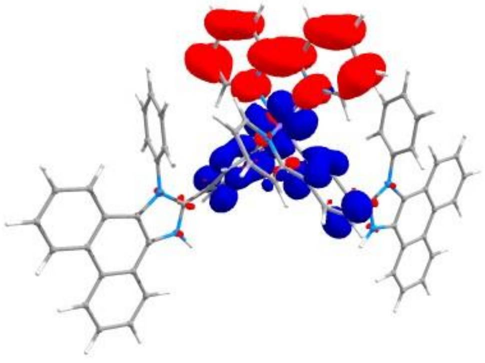

$S_0 \rightarrow S_1$

Donor	Acceptor			
	Ir	NN	N-C	N-C
Ir	0.019	0.282	0.046	0.046
NN	0.001	0.017	0.003	0.003
N-C	0.014	0.210	0.034	0.034
N-C	0.014	0.210	0.034	0.034

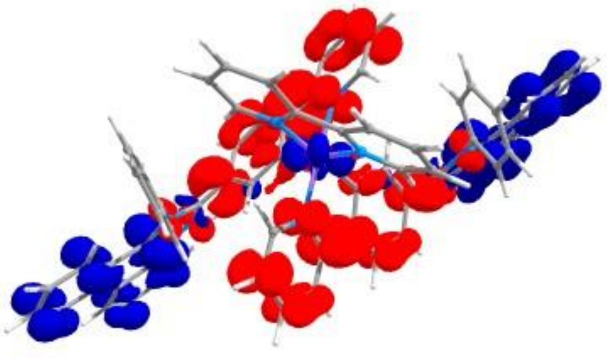

$S_0 \rightarrow S_2$

Donor	Acceptor			
	Ir	NN	N-C	N-C
Ir	0.001	0.001	0.020	0.020
NN	0.000	0.000	0.001	0.001
N-C	0.016	0.008	0.227	0.227
N-C	0.016	0.008	0.227	0.227

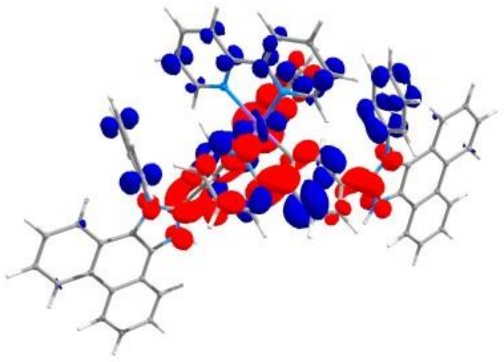

$S_0 \rightarrow S_3$

Donor	Acceptor			
	Ir	NN	N-C	N-C
Ir	0.002	0.001	0.034	0.034
NN	0.004	0.001	0.054	0.054
N-C	0.013	0.003	0.196	0.196
N-C	0.013	0.003	0.196	0.196

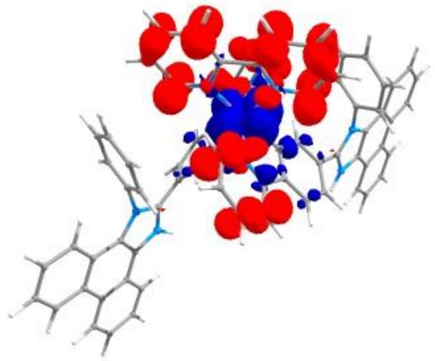

$S_0 \rightarrow S_4$

Donor	Acceptor			
	Ir	NN	N-C	N-C
Ir	0.007	0.127	0.115	0.115
NN	0.002	0.046	0.041	0.041
N-C	0.005	0.089	0.080	0.080
N-C	0.005	0.089	0.080	0.080

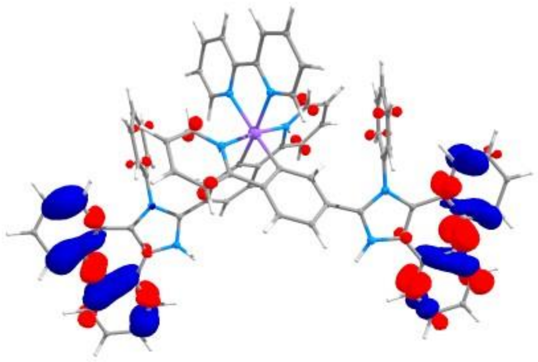

$S_0 \rightarrow S_5$

Donor	Acceptor			
	Ir	NN	N-C	N-C
Ir	0.000	0.000	0.008	0.008
NN	0.000	0.000	0.001	0.001
N-C	0.001	0.010	0.240	0.240
N-C	0.001	0.010	0.240	0.240

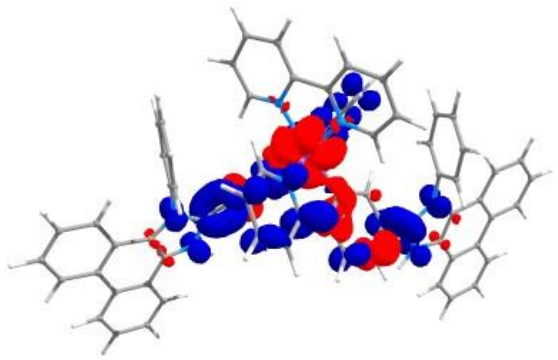

$T_1 \rightarrow S_0$

Donor	Acceptor			
	Ir	NN	N-C	N-C
Ir	0.010	0.001	0.013	0.013
NN	0.002	0.000	0.003	0.003
N-C	0.126	0.012	0.169	0.169
N-C	0.127	0.012	0.170	0.170

*Table S13. Partial protonation of complexes **1** and **2**: left – calculated absorption spectra for complexes **1** and **2** and their mono- and di-protonated forms, central and right: the decrease (blue) and increase (red) in electron density for absorption and emission transitions in mono-protonated forms.*

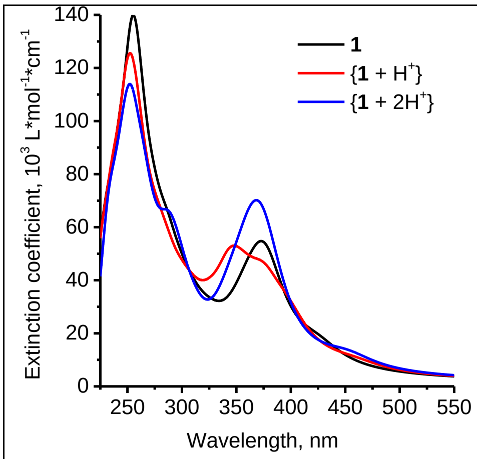	{**1**+1H$^+$} S_0-S_1<br>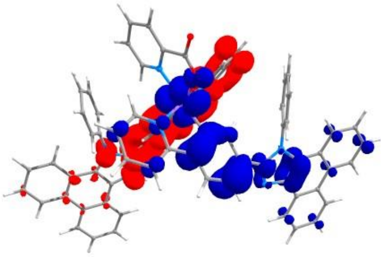	{**1**+1H$^+$} T_1-S_0<br>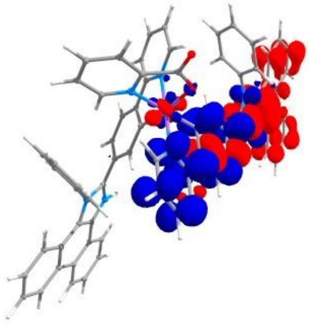
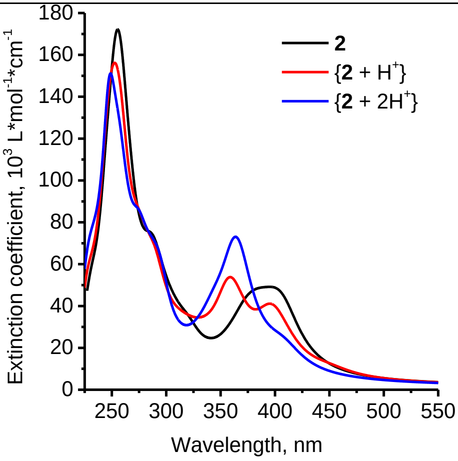	{**2**+1H$^+$} S_0-S_1<br>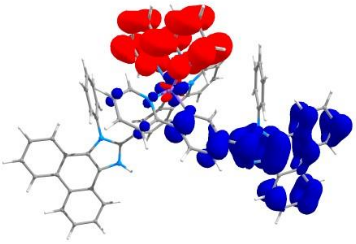	{**2**+1H$^+$} T_1-S_0<br>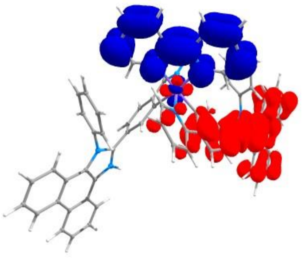

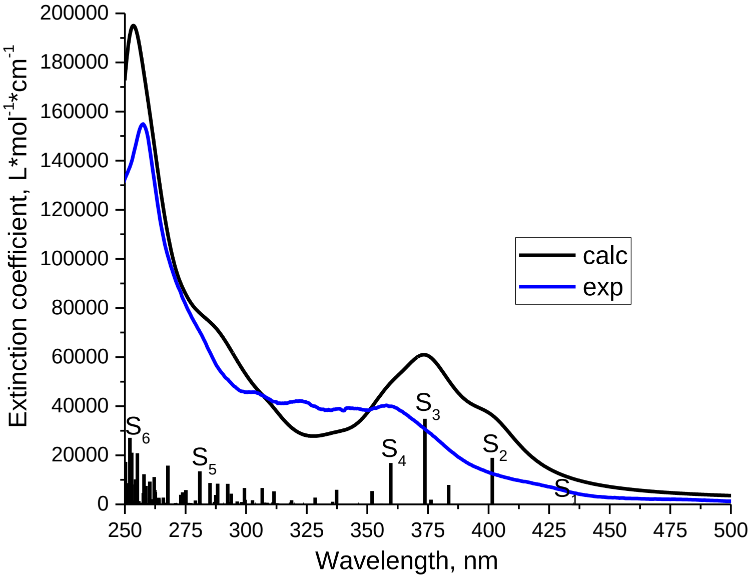

*Figure S22. Absorption spectra of complex {**3**-2H$^+$} (deprotonated at the **N^N** ligand form of **3**) in CH_3OH: experimental (blue) and calculated (black) lines with oscillator strengths of electronic transitions (bars).*

*Table S14. Experimental and calculated absorption maxima (λ), extinction coefficients (ε), oscillator strengths (f) {**3**-2H⁺}.*

Complex	λ_{abs}, nm (exp)	ε*10^{-3}, L*mol^{-1}*cm^{-1} (exp)	Transitions	λ_{abs}, nm (calc)	*f* (calc)	Contribution of main NTO pair in transition (%)
{**3**-2H$^+$}	258	155	S_0-S_6	252	0.37	29
	304	45	S_0-S_5	281	0.18	52
	357	40	S_0-S_4	360	0.23	88
			S_0-S_3	374	0.47	90
	420sh	8	S_0-S_2	402	0.26	95
	480sh	2	S_0-S_1	435	0.00	98

*Table S15. The decrease (blue) and increase (red) in electron density for most intensive electronic absorption transitions of {**3**-2H⁺}. The data for the corresponding interfragment charge transfer (IFCT) are given below the figures. Diagonal values represent intraligand transitions, off-diagonal values represent a charge transfer from "Donor" to "Acceptor".*

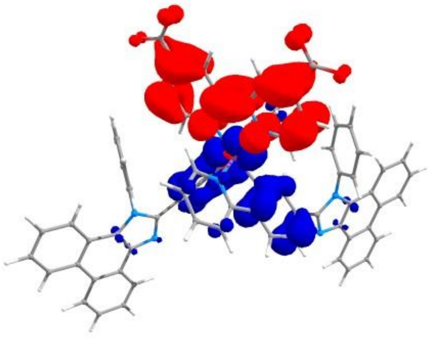

$S_0 \rightarrow S_1$

Donor	Acceptor			
	Ir	NN	N-C	N-C
Ir	0.013	0.323	0.002	0.002
NN	0.001	0.021	0.000	0.000
N-C	0.013	0.302	0.002	0.002
N-C	0.013	0.302	0.002	0.002

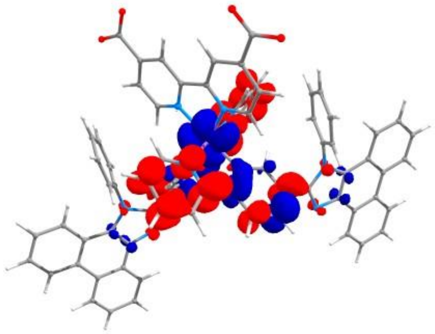

$S_0 \rightarrow S_2$

Donor	Acceptor			
	Ir	NN	N-C	N-C
Ir	0.014	0.001	0.150	0.150
NN	0.001	0.000	0.010	0.010
N-C	0.015	0.001	0.158	0.158
N-C	0.015	0.001	0.158	0.158

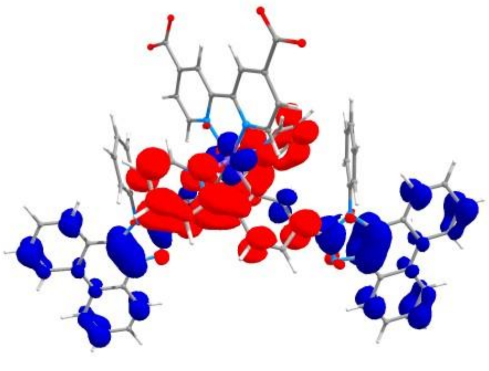

$S_0 \rightarrow S_3$

Donor	Acceptor			
	Ir	NN	N-C	N-C
Ir	0.004	0.000	0.042	0.042
NN	0.000	0.000	0.003	0.003
N-C	0.020	0.002	0.216	0.216
N-C	0.020	0.002	0.216	0.216

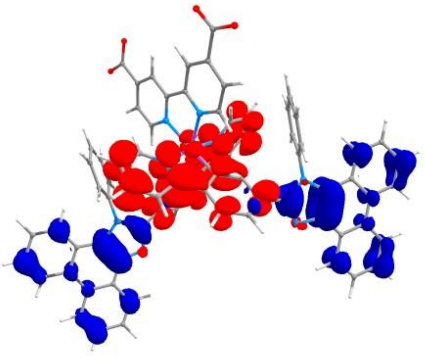

$S_0 \rightarrow S_4$

Donor	Acceptor			
	Ir	NN	N-C	N-C
Ir	0.000	0.000	0.004	0.004
NN	0.000	0.000	0.000	0.000
N-C	0.021	0.003	0.236	0.236
N-C	0.021	0.003	0.236	0.236

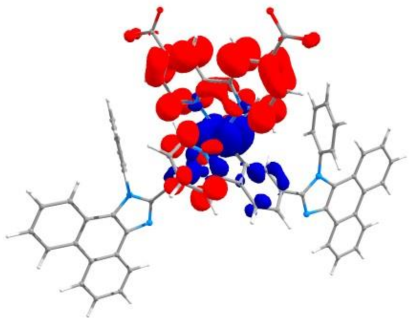

$S_0 \rightarrow S_5$

Donor	Acceptor			
	Ir	NN	N-C	N-C
Ir	0.010	0.240	0.070	0.070
NN	0.003	0.079	0.023	0.023
N-C	0.006	0.149	0.043	0.043
N-C	0.006	0.148	0.043	0.043

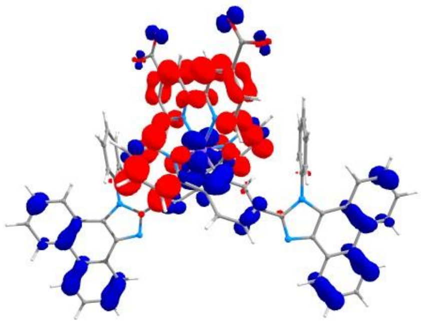

$S_0 \rightarrow S_6$

Donor	Acceptor			
	Ir	NN	N-C	N-C
Ir	0.003	0.037	0.031	0.031
NN	0.005	0.061	0.050	0.050
N-C	0.011	0.134	0.110	0.110
N-C	0.011	0.134	0.111	0.111

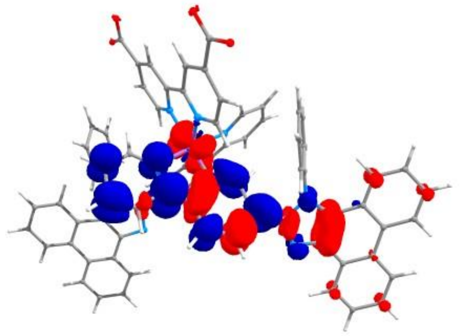

$T_1 \rightarrow S_0$

Donor	Acceptor			
	Ir	NN	N-C	N-C
Ir	0.008	0.001	0.004	0.031
NN	0.003	0.000	0.001	0.010
N-C	0.008	0.001	0.004	0.031
N-C	0.160	0.022	0.079	0.638

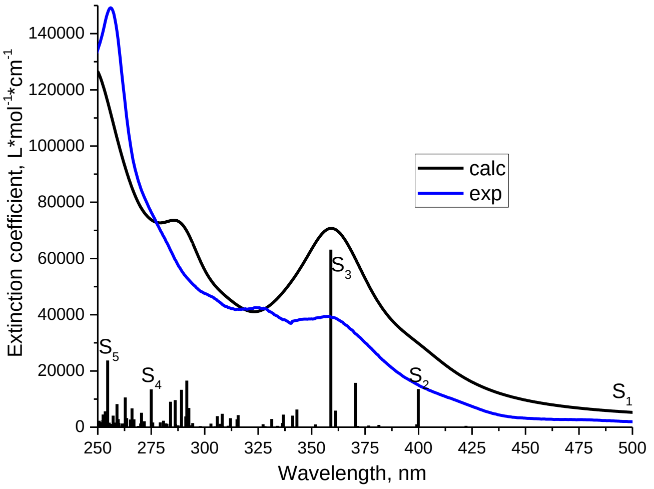

*Figure S23. Absorption spectra of complex {**3**+2H$^+$} in CH_3OH: experimental (blue) and calculated (black) lines with oscillator strengths of electronic transitions (bars).*

*Table S16 Experimental and calculated absorption maxima (λ), extinction coefficients (ε), oscillator strengths (f) {**3**+2H$^+$}.*

Complex	λ_{abs}, nm (exp)	ε*10^{-3}, L*mol^{-1}*cm^{-1} (exp)	Transitions	λ_{abs}, nm (calc)	*f* (calc)	Contribution of main NTO pair in transition (%)
{**3**+2H$^+$}	255	148	S_0-S_5	254	0.32	41
			S_0-S_4	275	0.18	36
	357	40	S_0-S_3	359	0.86	75
	420sh	9	S_0-S_2	400	0.18	88
	480sh	2	S_0-S_1	496	0.00	99

.

*Table S17. The decrease (blue) and increase (red) in electron density for most intensive electronic absorption transitions of {**3**+2H$^+$}. The data for the corresponding interfragment charge transfer (IFCT) are given below the figures. Diagonal values represent intraligand transitions, off-diagonal values represent a charge transfer from "Donor" to "Acceptor".*

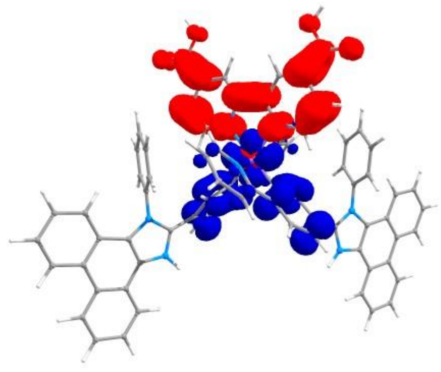

$S_0 \rightarrow S_1$

Donor	Acceptor			
	Ir	NN	N-C	N-C
Ir	0.016	0.357	0.002	0.002
NN	0.001	0.022	0.000	0.000
N-C	0.013	0.284	0.002	0.002
N-C	0.013	0.284	0.002	0.002

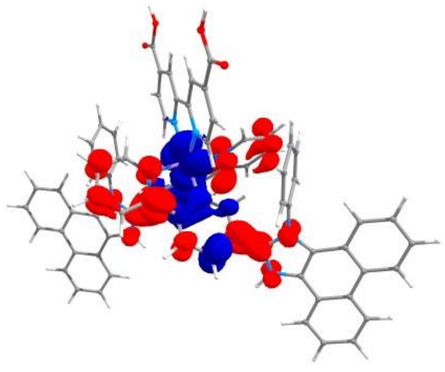

$S_0 \rightarrow S_2$

Donor	Acceptor			
	Ir	NN	N-C	N-C
Ir	0.014	0.033	0.171	0.171
NN	0.001	0.002	0.011	0.011
N-C	0.011	0.025	0.128	0.129
N-C	0.011	0.025	0.129	0.129

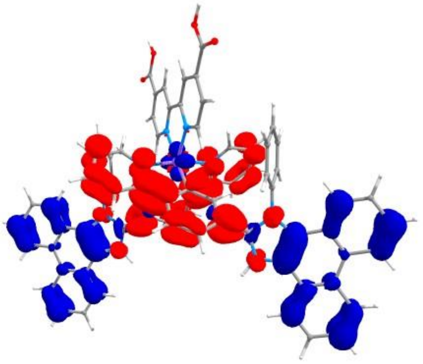

$S_0 \rightarrow S_3$

Donor	Acceptor			
	Ir	NN	N-C	N-C
Ir	0.001	0.000	0.016	0.016
NN	0.000	0.000	0.001	0.001
N-C	0.017	0.005	0.230	0.230
N-C	0.017	0.005	0.230	0.230

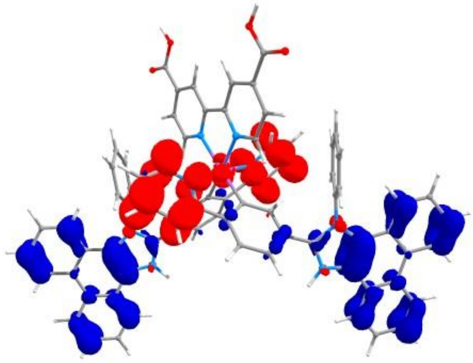

$S_0 \rightarrow S_4$

Donor	Acceptor			
	Ir	NN	N-C	N-C
Ir	0.003	0.007	0.052	0.052
NN	0.001	0.001	0.011	0.011
N-C	0.011	0.027	0.197	0.197
N-C	0.011	0.027	0.197	0.197

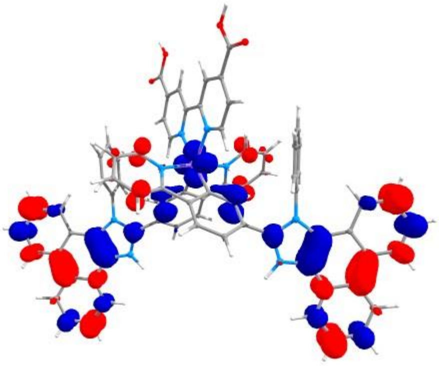

$S_0 \rightarrow S_5$

Donor	Acceptor			
	Ir	NN	N-C	N-C
Ir	0.000	0.000	0.029	0.029
NN	0.000	0.000	0.002	0.002
N-C	0.004	0.002	0.234	0.234
N-C	0.003	0.002	0.229	0.229

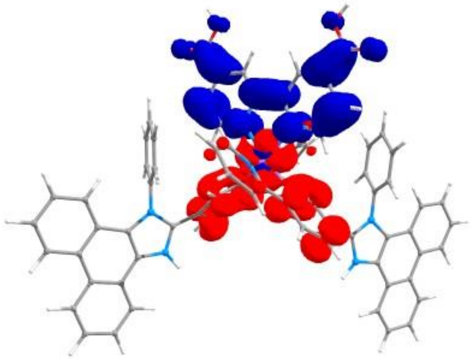

$T_1 \rightarrow S_0$

Donor	Acceptor			
	Ir	NN	N-C	N-C
Ir	0.017	0.001	0.014	0.014
NN	0.340	0.029	0.287	0.287
N-C	0.002	0.000	0.002	0.002
N-C	0.002	0.000	0.002	0.002

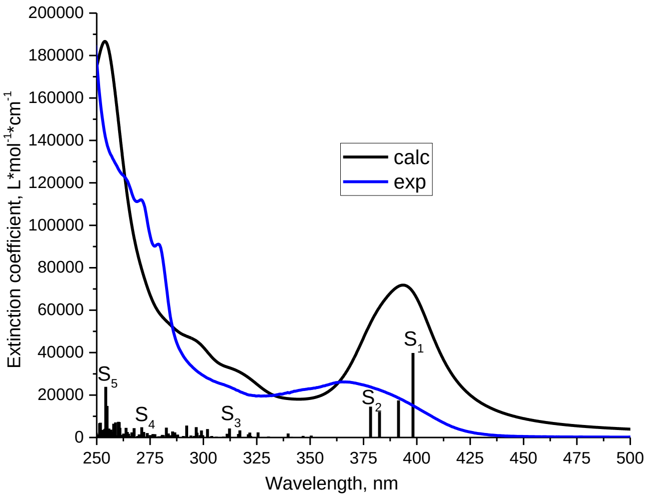

*Figure S24. Absorption spectra of **4** in water: experimental (blue) and calculated (black) lines with oscillator strengths of electronic transitions (bars).*

*Table S18. Experimental and calculated absorption maxima (λ), extinction coefficients (ε), oscillator strengths (f) **4**.*

Complex	λ_{abs}, nm (exp)	ε*10^{-3}, L*mol^{-1}*cm^{-1} (exp)	Transitions	λ_{abs}, nm (calc)	*f* (calc)	Contribution of main NTO pair in transition (%)
**4**	263sh	128	S_0-S_5	255	0.20	27
	271	117	S_0-S_4	271	0.07	36
	308sh	25	S_0-S_3	312	0.06	53
	367	27	S_0-S_2	378	0.20	97
			S_0-S_1	398	0.54	94

*Table S19. The decrease (blue) and increase (red) in electron density for most intensive electronic absorption transitions of **4**. The data for the corresponding interfragment charge transfer (IFCT) are given below the figures. Diagonal values represent intraligand transitions, off-diagonal values represent a charge transfer from "Donor" to "Acceptor".*

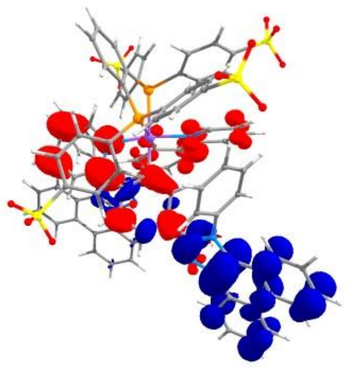

$S_0 \rightarrow S_1$

Donor	Acceptor			
	Ir	PP	N-C	N-C
Ir	0.000	0.000	0.005	0.002
PP	0.000	0.000	0.004	0.001
N-C	0.021	0.036	0.616	0.190
N-C	0.003	0.005	0.089	0.027

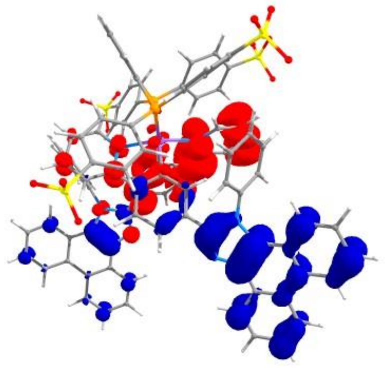

$S_0 \rightarrow S_2$

Donor	Acceptor			
	Ir	PP	N-C	N-C
Ir	0.000	0.000	0.000	0.002
PP	0.000	0.000	0.001	0.003
N-C	0.016	0.030	0.114	0.532
N-C	0.007	0.013	0.050	0.232

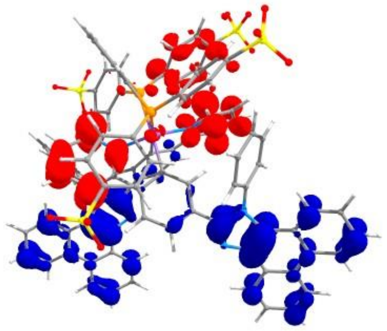

$S_0 \rightarrow S_3$

Donor	Acceptor			
	Ir	PP	N-C	N-C
Ir	0.001	0.009	0.010	0.012
PP	0.000	0.003	0.003	0.004
N-C	0.017	0.110	0.129	0.146
N-C	0.023	0.152	0.179	0.203

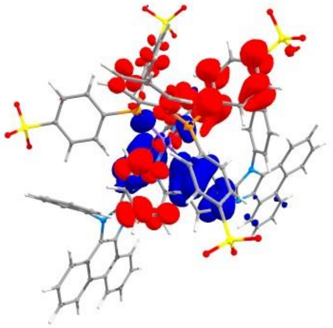

$S_0 \rightarrow S_4$

Donor	Acceptor			
	Ir	PP	N-C	N-C
Ir	0.001	0.019	0.007	0.008
PP	0.005	0.086	0.031	0.036
N-C	0.010	0.178	0.065	0.074
N-C	0.015	0.261	0.096	0.109

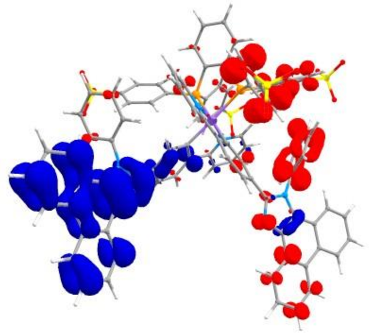

$S_0 \rightarrow S_5$

Donor	Acceptor			
	Ir	PP	N-C	N-C
Ir	0.000	0.009	0.012	0.006
PP	0.000	0.013	0.017	0.009
N-C	0.003	0.099	0.132	0.066
N-C	0.007	0.210	0.278	0.139

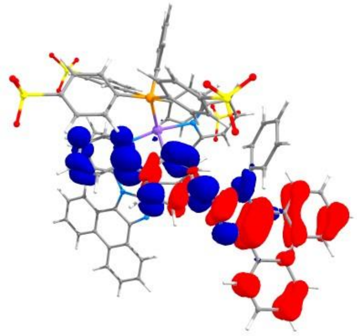

$T_1 \rightarrow S_0$

Donor	Acceptor			
	Ir	PP	N-C	N-C
Ir	0.000	0.000	0.000	0.014
PP	0.000	0.000	0.000	0.021
N-C	0.000	0.000	0.000	0.022
N-C	0.009	0.003	0.005	0.926

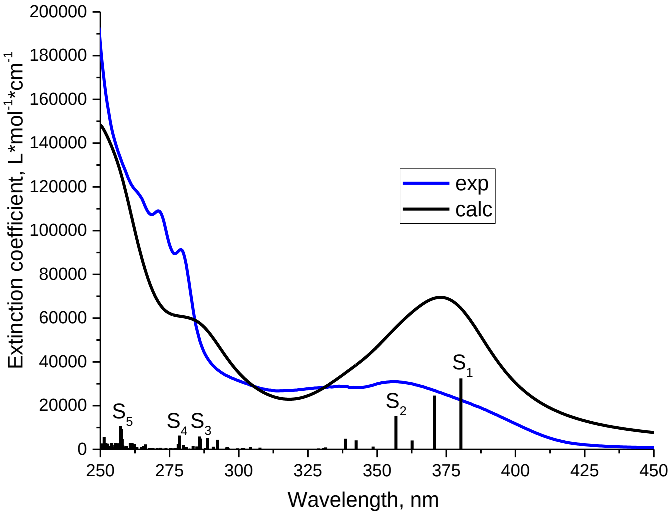

*Figure S25. Absorption spectra of {**4**+2H$^+$} in water: experimental (blue) and calculated (black) lines with oscillator strengths of electronic transitions (bars).*

*Table S20. Experimental and calculated absorption maxima (λ), extinction coefficients (ε), oscillator strengths (f) {**4**+2H$^+$}.*

Complex	λ_{abs}, nm (exp)	ε*10^{-3}, L*mol^{-1}*cm^{-1} (exp)	Transitions	λ_{abs}, nm (calc)	*f* (calc)	Contribution of main NTO pair in transition (%)
{**4**+2H$^+$}	264sh	117	S_0-S_5	257	0.19	28
	271	110	S_0-S_4	279	0.11	41
	279	91	S_0-S_3	286	0.10	58
	357	31	S_0-S_2	357	0.27	70
			S_0-S_1	380	0.57	92

*Table S21. The decrease (blue) and increase (red) in electron density for most intensive electronic absorption transitions of {**4**+2H$^+$}. The data for the corresponding interfragment charge transfer (IFCT) are given below the figures. Diagonal values represent intraligand transitions, off-diagonal values represent a charge transfer from "Donor" to "Acceptor".*

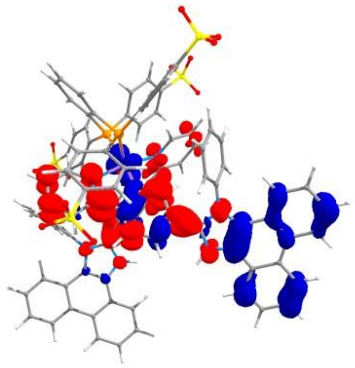

S_0→S_1

Donor	Acceptor			
	Ir	PP	N-C	N-C
Ir	0.001	0.002	0.059	0.029
PP	0.001	0.001	0.025	0.012
N-C	0.010	0.018	0.440	0.215
N-C	0.003	0.005	0.121	0.059

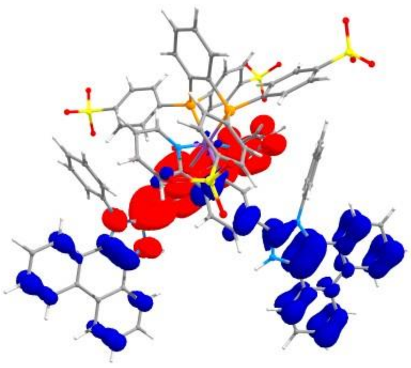

S_0→S_2

Donor	Acceptor			
	Ir	PP	N-C	N-C
Ir	0.001	0.001	0.017	0.040
PP	0.001	0.001	0.009	0.020
N-C	0.010	0.013	0.162	0.379
N-C	0.006	0.008	0.100	0.233

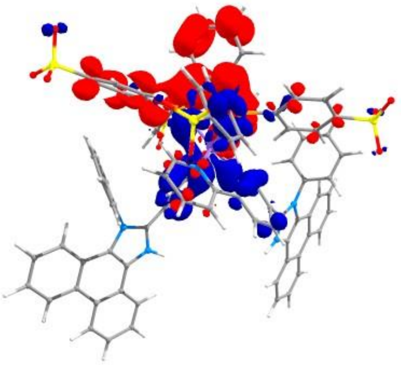

S$_0$→S$_3$

Donor	Acceptor			
	Ir	PP	N-C	N-C
Ir	0.002	0.034	0.015	0.006
PP	0.016	0.225	0.100	0.038
N-C	0.017	0.240	0.106	0.040
N-C	0.007	0.095	0.042	0.016

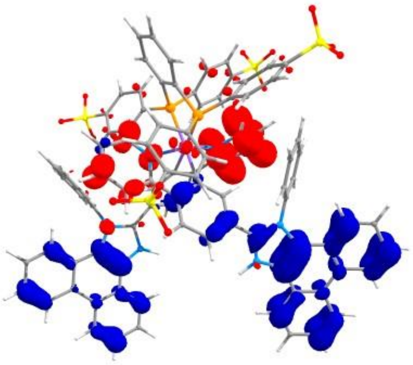

S$_0$→S$_4$

Donor	Acceptor			
	Ir	PP	N-C	N-C
Ir	0.002	0.013	0.008	0.025
PP	0.002	0.016	0.009	0.030
N-C	0.018	0.133	0.077	0.250
N-C	0.015	0.116	0.067	0.219

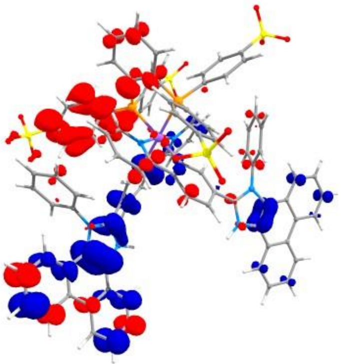

S$_0$→S$_5$

Donor	Acceptor			
	Ir	PP	N-C	N-C
Ir	0.000	0.006	0.005	0.009
PP	0.000	0.008	0.008	0.012
N-C	0.004	0.082	0.081	0.130
N-C	0.009	0.181	0.178	0.286

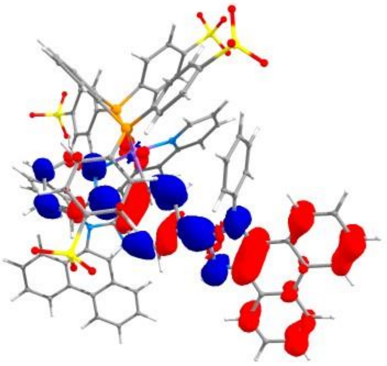

T$_1$→S$_0$

Donor	Acceptor			
	Ir	PP	N-C	N-C
Ir	0.000	0.001	0.013	0.000
PP	0.001	0.001	0.017	0.000
N-C	0.027	0.039	0.869	0.012
N-C	0.001	0.001	0.018	0.000
